# Initiation of Tocilizumab or Baricitinib Were Associated With Comparable Clinical Outcomes Among Patients Hospitalized With COVID-19 and Treated With Dexamethasone

**DOI:** 10.3389/fphar.2022.866441

**Published:** 2022-05-30

**Authors:** Carlos K. H. Wong, Kristy T. K. Lau, Ivan C. H. Au, Xi Xiong, Matthew S. H. Chung, Belle Y. C. Leung, Eric H. Y. Lau, Benjamin J. Cowling

**Affiliations:** ^1^ Department of Pharmacology and Pharmacy, LKS Faculty of Medicine, The University of Hong Kong, Hong Kong, Hong Kong SAR, China; ^2^ Department of Family Medicine and Primary Care, School of Clinical Medicine, LKS Faculty of Medicine, The University of Hong Kong, Hong Kong, Hong Kong SAR, China; ^3^ Laboratory of Data Discovery for Health Limited, Hong Kong, Hong Kong SAR, China; ^4^ WHO Collaborating Centre for Infectious Disease Epidemiology and Control, School of Public Health, LKS Faculty of Medicine, The University of Hong Kong, Hong Kong, Hong Kong SAR, China

**Keywords:** baricitinib, clinical improvement, COVID-19, dexamethasone, tocilizumab

## Abstract

**Objectives:** This retrospective cohort study aims to explore head-to-head clinical outcomes and complications associated with tocilizumab or baricitinib initiation among hospitalized COVID-19 patients receiving dexamethasone.

**Methods:** Among 10,445 COVID-19 patients hospitalized between January 21st 2020 and January 31st 2021 in Hong Kong, patients who had received tocilizumab (*n* = 165) or baricitinib (*n* = 76) while on dexamethasone were included. Primary study outcome was time to clinical improvement (at least one score reduction on WHO clinical progression scale). Secondary outcomes were disease progression, viral dynamics, in-hospital death, hyperinflammatory syndrome, and COVID-19/treatment-related complications. Hazard ratios (HR) of event outcomes were estimated using Cox regression models.

**Results:** The initiation of tocilizumab or baricitinib had no significant differences in time to clinical improvement (HR = 0.86, 95%CI 0.57-1.29, *p* = 0.459), hospital discharge (HR = 0.85, 95%CI 0.57-1.27, *p* = 0.418), recovery without the need for oxygen therapy (HR = 1.04, 95%CI 0.64-1.67, *p* = 0.883), low viral load (HR = 1.49, 95%CI 0.85-2.60, *p* = 0.162), and positive IgG antibody (HR = 0.97, 95%CI 0.61-1.54, *p* = 0.909). Time to viral clearance (HR = 1.94, 95%CI 1.01-3.73, *p* = 0.048) was shorter in the tocilizumab group with marginal significance, compared to that of baricitinib. Meanwhile, the two treatment modalities were not significantly different in their associated risks of in-hospital death (HR = 0.63, 95%CI 0.29-1.35, *p* = 0.233), severe liver injury (HR = 1.15, 95%CI 0.43-3.08, *p* = 0.778), acute renal failure (HR = 2.33, 95%CI 0.61-8.82, *p* = 0.213), hyperinflammatory syndrome (HR = 2.32, 95%CI 0.87-6.25, *p* = 0.091), thrombotic and bleeding events (HR = 1.39, 95%CI 0.32-6.00, *p* = 0.658), and secondary infection (HR = 2.97, 95%CI 0.62-14.31, *p* = 0.173).

**Conclusion:** Among hospitalized patients with moderate-to-severe COVID-19 on background dexamethasone, the initiation of tocilizumab or baricitinib had generally comparable effects on time to clinical improvement, hospital discharge, recovery, low viral load, and positive IgG antibody; risks of in-hospital death, hepatic and renal complications, hyperinflammatory syndrome, thrombotic and bleeding events, and secondary infection. On the other hand, tocilizumab users might achieve viral clearance slightly faster than baricitinib users. Further studies and clinical trials are needed to confirm our findings regarding the evaluation of tocilizumab and baricitinib in COVID-19 patients with different disease severities, at varying stages or timing of drug initiation, and considering the concomitant use of other therapeutics.

## Introduction

In the development of Coronavirus Disease 2019 (COVID-19), increased risks of acute respiratory distress syndrome (ARDS), shock, and multiorgan dysfunction have been attributed to the second phase of hyperinflammation or “cytokine storm” (Alunno et al., 2021; Nissen et al., 2021). Numerous drugs have been repurposed to target the various viral and host immune response mechanisms responsible for the infectivity and severity of COVID-19, for instance, antivirals and immunomodulators (Feuillet et al., 2021). Remdesivir is an antiviral medication with potential benefits in facilitating recovery and survival of COVID-19 patients who are on low-flow oxygen therapy, and possibly those breathing ambient air (Beigel et al., 2020; Infectious Diseases Society of America, 2022; National Institutes of Health, 2022). Meanwhile, the corticosteroid dexamethasone has been shown to significantly reduce mortality and disease progression among patients on supplemental oxygen or mechanical ventilation, hence being recommended in current guidelines (The RECOVERY Collaborative Group, 2020; Alunno et al., 2021; World Health Organization, 2021; Infectious Diseases Society of America, 2022; National Institutes of Health, 2022).

As one of the major cytokines regulating inflammatory response in COVID-19, serum interleukin-6 (IL-6) has been observed to correlate with mortality, and proposed as a biomarker predictive of disease severity (Liu et al., 2020; McElvaney et al., 2021). Accordingly, inhibition of IL-6 signaling has been suggested as a means of reducing infection-related complications and organ damage *via* an attenuation of the cytokine cascade; where tocilizumab is a monoclonal antibody directed against the IL-6 receptor (Ingraham et al., 2020; Liu et al., 2020; McElvaney et al., 2021). Randomized controlled trials of tocilizumab have reported conflicting results in COVID-19 patients, with some demonstrating benefits of survival, hospital discharge, and lowering the need for mechanical ventilation; while others have failed to find significant differences compared to control (Salama et al., 2020; Stone et al., 2020; Hermine et al., 2021; RECOVERY Collaborative Group, 2021; Rosas et al., 2021; Salvarani et al., 2021; Soin et al., 2021; The REMAP-CAP Investigators, 2021; Veiga et al., 2021). Heterogeneity in the study population may be responsible for these observations, namely disease severity and mortality risk of patients at baseline, level of respiratory support required, drug regimens of standard care, and any concomitant use of other potential therapeutics. Overall, meta-analyses of clinical trials and observational studies have contributed to current guidelines recommending the use of tocilizumab in severe COVID-19 patients who are on oxygen therapy or within 24 h of intubation, with evidence of systemic inflammation, and preferentially with concomitant corticosteroids, in view of the significant reduction in mortality and ventilatory support for these patients (Chen et al., 2021a; Klopfenstein et al., 2021; The W. H. O. Rapid Evidence Appraisal for Covid-Therapies Working Group, 2021; Tleyjeh et al., 2021; Zhao et al., 2021; Infectious Diseases Society of America, 2022; National Institutes of Health, 2022).

Another therapeutic approach for managing COVID-19 is targeting the Janus kinase/signal transducer and activator of transcription (JAK/STAT) pathway, which mediates the signaling of multiple pro-inflammatory cytokines, including IL-6 (Richardson et al., 2020; Spinelli et al., 2020; Solimani et al., 2021). Consequently, it is hoped that JAK inhibition would help restrain the “cytokine storm”, suppress hyperinflammation and progression to ARDS (Richardson et al., 2020; Spinelli et al., 2020; Solimani et al., 2021). In addition to anti-inflammatory properties, baricitinib is a JAK inhibitor with potential antiviral effects, inhibiting AP2-associated kinase 1 (AAK1) and cyclin G-associated kinase (GAK) involved in SARS-CoV-2 endocytosis, i.e. viral infection of host cells (Jorgensen et al., 2020; Richardson et al., 2020; Spinelli et al., 2020). Results from ACTT-2 trial suggest that combined use of baricitinib and remdesivir would hasten recovery of COVID-19 patients compared to remdesivir alone, and those of COV-BARRIER trial suggest a survival benefit, both particularly evident among patients on high-flow oxygen therapy or non-invasive ventilation (Kalil et al., 2021; Marconi et al., 2021). With further evidence identifying lower risks of mortality and mechanical ventilation, baricitinib is recommended for COVID-19 patients on supplemental oxygen but not invasive ventilation, in the presence of systemic inflammation, and with concomitant remdesivir or dexamethasone (Alunno et al., 2021; Chen et al., 2021b; Infectious Diseases Society of America, 2022; National Institutes of Health, 2022).

In response to a lack of head-to-head comparison between tocilizumab and baricitinib (Infectious Diseases Society of America, 2022; National Institutes of Health, 2022), this retrospective cohort study aims to evaluate the safety and efficacy of these two treatment modalities in COVID-19 patients. Based on current guidelines, this study will focus on comparing tocilizumab against the use of baricitinib among dexamethasone users, on suggested outcomes of mortality, viral dynamics, disease progression and recovery (Marshall et al., 2020), complications of COVID-19 and risk of secondary infection.

## Materials and Methods

### Ethics

The study protocol was approved by the Institutional Review Board of the University of Hong Kong/Hospital Authority Hong Kong West Cluster (Reference No. UW 20-493). Given the extraordinary nature of the COVID-19 pandemic, individual patient informed consent was not required for this retrospective cohort study using anonymized data.

### Data Source

This territory-wide retrospective cohort study was conducted in the Hong Kong Special Administrative Region, China, where all confirmed cases of COVID-19 were treated at local public hospitals managed under the Hospital Authority. Electronic medical records of all admitted patients with COVID-19 confirmed by positive reverse transcription polymerase chain reaction (RT-PCR) test were collected from the Hospital Authority database for the study period from January 21st, 2020 to January 31st, 2021, including patient demographics, deaths, diagnoses, procedures, drug prescription and dispensing history, and laboratory test results. The Hospital Authority database has been used extensively for the evaluation of drug therapies for COVID-19 (Wong et al., 2021a; Wong et al., 2021b; Wong et al., 2022a; Wong et al., 2022b). The follow-up period lasted until April 30th, 2021.

According to the latest Hong Kong Hospital Authority Interim Drug Treatment Handbook for COVID-19, dexamethasone, tocilizumab, and baricitinib were considered as anti-inflammatory treatment options for COVID-19 patients (Department of Pharmacy, 2021). Daily dose should be 6 mg of dexamethasone orally or intravenously for up to 10 days, which was recommended for hospitalized COVID-19 patients with pneumonia, and those who required supplemental oxygen or invasive mechanical ventilation. Intravenous tocilizumab (4–8 mg per kilogram of body weight, to a maximum of 800 mg per dose for adults), an unlicensed treatment for COVID-19, could be administered at the discretion of physicians for severe patients with evidence of cytokine release syndrome. Oral baricitinib of 4 mg per day for up to 14 days or until hospital discharge (whichever came first) could be used in severe or critically ill patients at the discretion of physicians; and in combination with remdesivir, based on the observed clinical benefits of their combined use in the ACTT-2 trial (Kalil et al., 2021).

### Study Population

In an attempt to eliminate selection and immortal time biases, “active comparator, new user” study design (Lund et al., 2015) was adopted to identify COVID-19 patients who had initiated baricitinib (denoted as “baricitinib”) or tocilizumab (denoted as “tocilizumab”) among those treated with dexamethasone. The distribution of timing of dexamethasone, and tocilizumab or baricitinib initiation by the two treatment groups are illustrated in Supplementary Figure S1. The inclusion of eligible patients for this study was depicted in Supplementary Figure S2. The index date was defined as the first tocilizumab or baricitinib dispensing date. Patients were observed from the index date to in-hospital death, hospital discharge, treatment crossover (i.e. patients crossing over from baricitinib to tocilizumab treatment, or vice versa), or censored on April 30th, 2021, whichever came first.

### Baseline Covariates

Baseline covariates of patients consisted of age, sex, pre-existing comorbidities, anticoagulant and antiplatelet use, treatments received before the index date, clinical severity defined by the WHO clinical progression scale (CPS) (Marshall et al., 2020), risk of disease progression determined by Sequential Organ Failure Assessment (SOFA) score, and laboratory parameters. Laboratory parameters included white blood cell count, neutrophil count, lymphocyte count, platelet count, lactate dehydrogenase (LDH), creatine kinase (CK), total bilirubin, c-reactive protein (CRP), ferritin, cycle threshold (Ct) value, estimated glomerular filtration rate (eGFR), alkaline phosphatase (ALP), alanine aminotransferase (ALT), and hemoglobin. Details of dexamethasone, tocilizumab, baricitinib, and any concomitant remdesivir treatments were documented, namely time from admission to drug initiation, duration of use, cumulative dosage, and route of administration. Other treatments received on or before the index date were recorded, namely interferon-β-1b, ribavirin, other systemic steroids (hydrocortisone, prednisolone, or methylprednisolone), extracorporeal membrane oxygenation (ECMO), invasive mechanical ventilation, dialysis, and intensive care unit (ICU) admission during hospitalization. Diagnoses of severe liver injury, acute renal failure (ARF), hyperinflammatory syndrome (as defined by Webb et al.; including macrophage activation, hematological dysfunction, coagulopathy, and hepatic inflammation) (Webb et al., 2020), and thrombotic and bleeding events on or before the index date were also identified.

### Outcome Measures

For the primary study outcome, patients were observed from the index date until clinical improvement (defined as reduction on the WHO CPS by at least one score). Secondary outcomes included time to hospital discharge; recovery without the need for oxygen therapy; viral clearance (first negative PCR result); low viral load (Ct value ≥ 35 cycles); positive antibody against SARS-CoV-2 (first positive IgG antibody); composite outcome of in-hospital death, invasive mechanical ventilation, or ICU admission; in-hospital death; severe liver injury; ARF; hyperinflammatory syndrome; thrombotic and bleeding events; and secondary infection (of herpes simplex virus; methicillin-resistant *Staphylococcus aureus*; pneumoniae, influenza, and respiratory virus; and *Strongyloides stercoralis*, of which concerns about strongyloidiasis have been raised regarding the combined use of dexamethasone and tocilizumab (National Institutes of Health, 2022)).

Other outcomes were hospital length of stay (LOS) for discharged patients (estimating from hospital admission to discharge); changes in clinical status and average WHO CPS score over follow-up; estimation of cumulative direct medical costs incurred by patients from baseline to day-90; and changes in laboratory parameters from baseline to day-30. Direct medical costs comprised those associated with drug use and various healthcare services in the local setting (Supplementary Table S1).

### Statistical Analyses

Multiple imputations by chained equations were performed in adequately dealing with missing data of laboratory parameters (Supplementary Table S2) using other observed baseline covariates. Laboratory parameters were imputed 20 times and then used to generate multiple-imputation linear predictions by applying Rubin’s combination rules (Leyrat et al., 2019). Propensity scores (PS) of all patients were calculated by performing multivariable logistic regression adjusting for the baseline covariates aforementioned. Inverse probability of treatment weighting (IPTW) was used to equilibrate the baseline covariates of patients in tocilizumab and baricitinib groups. Extreme weights (e.g. 1st and 99th percentiles) were truncated to obtain a better balance between groups (Desai and Franklin, 2019). Such balance was further assessed using absolute standardized mean difference (SMD) before and after PS weighting, where SMD <0.2 would imply an optimal balance between treatment groups (Austin, 2009).

Cox hazard proportional regression models weighted by IPTW were applied to compare the risks of event outcomes between treatment groups. Hazard ratios (HR) with corresponding 95% confidence intervals (CI) were computed. Treatment effects were evaluated for continuous outcomes by linear regression using IPTW as the specified weight.

Sensitivity analyses were conducted by: 1) removing hospital discharge as censoring; 2) examining study outcomes for at most 90 days of follow-up; and 3) complete-case approach using IPTW. Meanwhile, PS and IPTW were re-calculated for the following subgroup analyses: age; sex; timing of drug initiation; route of administration of dexamethasone; dosage of dexamethasone, tocilizumab, and baricitinib; receipt of concomitant remdesivir, interferon-β-1b, and ribavirin; on supplemental oxygen but not mechanical ventilation; need for invasive mechanical ventilation or ECMO, and ICU admission.

All data management and statistical analyses were performed using STATA version 16.0 (StataCorp LP, College Station, TX). A *p*-value <0.05 was considered statistically significant.

## Results

Among 10,445 patients with COVID-19 admitted to hospital between January 21st, 2020 and January 31st, 2021, 1,544 patients were administered dexamethasone orally or intravenously during hospitalization (Supplementary Figure S2). In this cohort, 241 patients (15.6%) had also initiated tocilizumab or baricitinib, of whom 165 (68.5%) patients were given tocilizumab, and 76 (31.5%) patients were given baricitinib. Remdesivir was administered concomitantly in the vast majority of baricitinib users (89.5%), in accordance with current guidelines.

Baseline characteristics of patients of the two treatment groups before and after PS weighting are listed in Table 1. After multiple imputation and weighting, PS distribution of the two groups highly overlapped (Supplementary Figure S3). The mean (standard deviation) age of tocilizumab and baricitinib groups were 67.5 (11.7) and 67.4 (12.8) years respectively, with 67.7 and 66.0% male. Clinical severity of COVID-19, SOFA score, the presence of pre-existing comorbidities (predominantly hypertension and diabetes), anticoagulant and antiplatelet use, treatments received during hospitalization, and laboratory parameters were comparable between the two groups. Overall, baseline characteristics of patients were well balanced with all SMDs <0.2.

**TABLE 1:** Baseline characteristics of hospitalized patients with COVID-19 in tocilizumab and baricitinib groups after multiple imputation and propensity score weighting.

Baseline Characteristics	Before Weighting					After Weighting				
	Tocilizumab (*n* = 165)		Baricitinib (*n* = 76)		SMD	Tocilizumab (*n* = 165)		Baricitinib (*n* = 76)		SMD
	N/Mean	%/SD	N/Mean	%/SD		%/Mean	%/SD	N/Mean	%/SD	
Age, years^a^	67.3	12.2	67.1	12.9	0.02	67.5	11.7	67.4	12.8	0.01
<65	61	(37.0%)	27	(35.5%)	0.03	(37.9%)	—	(32.3%)	—	0.12
≥65	104	(63.0%)	49	(64.5%)	—	(62.1%)	—	(67.7%)	—	—
Sex	—	—	—	—	0.10	—	—		—	0.04
Male	106	(64.2%)	45	(59.2%)	—	(67.7%)	—	(66.0%)	—	—
Female	59	(35.8%)	31	(40.8%)	—	(32.3%)	—	(34.0%)	—	—
Time of treatment initiation	—	—	—	—	0.29	—	—	—	—	0.10
Before 2021-02-01	162	(98.2%)	70	(92.1%)	—	(97.5%)	—	(95.7%)	—	—
On or after 2021-02-01	3	(1.8%)	6	(7.9%)	—	(2.5%)	—	(4.3%)	—	—
Pre-existing comorbidities										
Charlson Comorbidity Index^a^ ^,^ ^b^	5.9	2.4	5.0	2.0	0.37	5.7	2.1	5.3	2.1	0.19
1-4	54	(32.9%)	30	(39.5%)	0.28	(28.5%)	—	(35.7%)	—	0.18
5-6	55	(33.5%)	30	(39.5%)	—	(42.5%)	—	(41.6%)	—	—
7-14	55	(33.5%)	16	(21.1%)	—	(29.1%)	—	(22.7%)	—	—
Diabetes mellitus	103	(62.4%)	40	(52.6%)	0.20	(61.9%)	—	(54.8%)	—	0.14
Hypertension	124	(75.2%)	55	(72.4%)	0.06	(75.4%)	—	(70.8%)	—	0.10
Liver disease	33	(20.0%)	5	(6.6%)	0.48	(15.7%)	—	(10.3%)	—	0.10
Chronic lung disease	43	(26.1%)	7	(9.2%)	0.40	(19.2%)	—	(12.1%)	—	0.16
Chronic heart disease	48	(29.1%)	16	(21.1%)	0.45	(27.0%)	—	(22.0%)	—	0.19
Chronic kidney disease	57	(34.5%)	11	(14.5%)	0.19	(26.4%)	—	(22.1%)	—	0.12
Long-term medications										
Anticoagulant	136	(82.4%)	71	(93.4%)	0.34	(86.8%)	—	(91.6%)	—	0.16
Antiplatelet	54	(32.7%)	10	(13.2%)	0.48	(23.9%)	—	(16.3%)	—	0.19
Treatment performed prior to baseline										
Tocilizumab	165	(100.0%)	0	(0.0%)	NA	(100.0%)	—	(0.0%)	—	NA
Time from admission to tocilizumab initiation, days^a^	5.9	5.2	NA	NA	NA	5.8	5.0	NA	NA	NA
Duration of use of tocilizumab, days^a^	1.1	0.3	NA	NA	NA	1.1	0.2	NA	NA	NA
Cumulative dosage of tocilizumab, mg^a^	483.1	122.6	NA	NA	NA	476.7	110.9	NA	NA	NA
Baricitinib	0	(0.0%)	76	(100.0%)	NA	(0.0%)	—	(100.0%)	—	NA
Time from admission to baricitinib initiation, days^a^	NA	NA	8.1	8.0	NA	NA	NA	7.7	8.3	NA
Duration of use of baricitinib, days^a^	NA	NA	7.3	5.0	NA	NA	NA	7.2	4.8	NA
Cumulative dosage of baricitinib, mg^a^	NA	NA	24.1	19.3	NA	NA	NA	24.4	19.0	NA
Dexamethasone	165	(100.0%)	76	(100.0%)	NA	(100.0%)	—	(100.0%)	NA	—
Time from admission to dexamethasone initiation, days^a^	2.7	3.0	3.5	3.6	0.26	2.6	2.8	3.2	3.3	0.19
Duration of use of dexamethasone, days^a^	17.5	17.6	14.9	13.8	0.16	16.1	16.2	17.4	17.0	0.08
Cumulative dosage of dexamethasone, mg^a^	113.7	116.5	92.7	72.7	0.20	105.7	110.4	106.5	85.9	0.01
Administration route of dexamethasone										
Oral	12	(7.3%)	13	(17.1%)	0.30	(10.7%)	—	(16.9%)	—	0.18
Intravenous injection	153	(92.7%)	63	(82.9%)	—	(89.3%)	—	(83.1%)	—	
Dosage of dexamethasone										
Up to 6 mg daily	62	(37.6%)	31	(40.8%)	0.07	(41.9%)	—	(37.3%)	—	0.09
More than 6 mg daily	103	(62.4%)	45	(59.2%)	—	(58.1%)	—	(62.7%)	—	—
Remdesivir	25	(15.2%)	68	(89.5%)	2.23	(12.6%)	—	(93.5%)	—	NA
Time from admission to remdesivir initiation, days^a^	5.3	3.7	3.7	3.8	0.43	6.0	3.3	3.4	3.8	NA
Duration of use of remdesivir, days^a^	4.5	1.5	5.0	2.4	0.25	4.3	1.3	4.8	2.0	NA
Cumulative dosage of remdesivir, mg^a^	502.8	231.1	698.6	274.8	0.75	534.0	187.1	676.6	222.7	NA
Interferon-β-1b	148	(89.7%)	62	(81.6%)	0.23	(90.2%)	—	(85.6%)	—	0.14
Ribavirin	57	(34.5%)	29	(38.2%)	0.08	(32.3%)	—	(38.2%)	—	0.13
Other systemic steroid	10	(6.1%)	1	(1.3%)	0.25	(4.3%)	—	(4.3%)	—	0.00
ECMO	0	(0.0%)	0	(0.0%)	NA	(0.0%)	—	(0.0%)	—	NA
Invasive mechanical ventilation	21	(12.7%)	11	(14.5%)	0.05	(10.0%)	—	(9.2%)	—	0.03
Dialysis	1	(0.6%)	0	(0.0%)	NA	(0.9%)	—	(0.0%)	—	NA
ICU admission	91	(55.2%)	44	(57.9%)	0.06	(59.2%)	—	(56.7%)	—	0.05
Clinical severity by WHO Clinical Progression Scale										
Score (range 0–10)^a^	5.5	1.3	5.4	1.1	0.08	5.5	1.2	5.4	1.1	0.11
No oxygen therapy (Score 4)	47	(28.5%)	25	(32.9%)	0.10	(28.3%)	—	(33.1%)	—	0.11
Supplemental oxygen without ventilation (Score 5-6)	99	(60.0%)	43	(56.6%)	—	(63.0%)	—	(59.7%)	—	—
Mechanical ventilation (Score 7-9)	19	(11.5%)	8	(10.5%)	—	(8.7%)	—	(7.3%)	—	—
SOFA score (range 0–24)^a^	7.5	1.4	7.6	1.4	0.08	7.7	1.4	7.6	1.6	0.02
Severe liver injury	3	(1.8%)	0	(0.0%)	NA	(1.3%)	—	(0.0%)	—	NA
Acute renal failure	1	(0.6%)	0	(0.0%)	NA	(0.4%)	—	(0.0%)	—	NA
Hyperinflammatory syndrome	147	(89.1%)	62	(81.6%)	0.21	(86.1%)	—	(84.9%)	—	0.03
Thrombotic and bleeding events	24	(14.5%)	62	(81.6%)	1.54	(31.9%)	—	(37.4%)	—	0.12

Note: ECMO, extracorporeal membrane oxygenation; ICU, intensive care unit; NA, not applicable; SD, standard deviation; SMD, standardized mean difference; SOFA, Sequential Organ Failure Assessment.

a Age, Charlson Comorbidity Index, clinical severity, cumulative dosage, duration of use of dosage, and time from admission to treatment initiation are presented in mean ± SD.

b The calculation of Charlson Comorbidity Index does not include Acquired Immune Deficiency Syndrome (AIDS) SMD, of <0.2 indicates covariate balance between tocilizumab and baricitinib groups.

The median follow-up period of this study cohort was 21 days, and the incidence rates of outcome events were detailed in Supplementary Table S3. No significant differences were identified between tocilizumab and baricitinib groups in time to clinical improvement (HR = 0.86, 95%CI 0.57-1.29, *p* = 0.459), hospital discharge (HR = 0.85, 95%CI 0.57-1.27, *p* = 0.418), recovery (HR = 1.04, 95%CI 0.64-1.67, *p* = 0.883), low viral load (HR = 1.49, 95%CI 0.85-2.60, *p* = 0.162), and positive IgG antibody (HR = 0.97, 95%CI 0.61-1.54, *p* = 0.909). Tocilizumab users had a shorter time to viral clearance (HR = 1.94, 95%CI 1.01-3.73, *p* = 0.048) of marginal significance, compared to baricitinib users (Table 2). In addition, no significant differences were observed in the risks of the composite outcome (HR = 0.96, 95%CI 0.47-2.00, *p* = 0.922), in-hospital death (HR = 0.63, 95%CI 0.29-1.35, *p* = 0.233), severe liver injury (HR = 1.15, 95%CI 0.43-3.08, *p* = 0.778), ARF (HR = 2.33, 95%CI 0.61-8.82, *p* = 0.213), hyperinflammatory syndrome (HR = 2.32, 95%CI 0.87-6.25, *p* = 0.091), secondary infection (HR = 2.97, 95%CI 0.62-14.31, *p* = 0.173), and thrombotic and bleeding events (HR = 1.39, 95%CI 0.32-6.00, *p* = 0.658) associated with tocilizumab or baricitinib initiation. Results of various sensitivity and subgroup analyses showed similar trends, and were generally comparable to those of main analysis (Supplementary Tables S4, S5).

**TABLE 2:** Comparison of clinical improvement on WHO clinical progression scale, hospital discharge, recovery, viral dynamics, clinical deterioration, in-hospital death, severe liver injury, acute renal failure, hyperinflammation syndrome, secondary infection, and thrombotic and bleeding events between tocilizumab and baricitinib groups.

Outcomes	Before Weighting		After Weighting		
	Tocilizumab	Baricitinib		Tocilizumab *vs*. baricitinib	
	% (N)	% (N)	HR^a^	95% CI	*p*-value
Clinical improvement on WHO clinical progression scale by ≥ 1 score	84.8% (165)	81.6% (76)	0.86	(0.57, 1.29)	0.459
Hospital discharge (score ≤ 3)	80.6% (165)	78.9% (76)	0.85	(0.57, 1.27)	0.418
Recovery (score ≤ 4)	75.4% (118)	68.6% (51)	1.04	(0.64, 1.67)	0.883
Viral clearance (first negative PCR result)	47.7% (155)	33.3% (72)	1.94	(1.01, 3.73)	0.048
Low viral load (Ct value ≥ 35)	46.8% (156)	35.2% (71)	1.49	(0.85, 2.60)	0.162
IgG antibody	85.5% (138)	84.9% (53)	0.97	(0.61, 1.54)	0.909

Outcomes	% (N)	% (N)	HR^b^	95% CI	*p*-value
In-hospital death or invasive mechanical ventilation (score ≥ 7) or intensive care unit admission	33.6% (137)	27.3% (66)	0.96	(0.47, 2.00)	0.922
In-hospital death (score = 10)	17.6% (165)	19.7% (76)	0.63	(0.29, 1.35)	0.233
Severe liver injury	18.5% (162)	13.2% (76)	1.15	(0.43, 3.08)	0.778
Acute renal failure	11.6% (164)	5.3% (76)	2.33	(0.61, 8.82)	0.213
Hyperinflammatory syndrome	88.9% (18)	64.3% (14)	2.32	(0.87, 6.25)	0.091
Secondary infection	7.4% (163)	2.6% (76)	2.97	(0.62, 14.31)	0.173
Thrombotic and bleeding events	10.6% (141)	4.9% (41)	1.39	(0.32, 6.00)	0.658

Note: CI, confidence interval; Ct = cycle threshold; HR, hazard ratio; IgG = immunoglobulin G; PCR, polymerase chain reaction.

a HR > 1 (or <1) indicates that tocilizumab use was associated with better (worse) clinical improvement, earlier (later) hospital discharge or recovery compared to that of baricitinib.

b HR > 1 (or <1) indicates that tocilizumab use was associated with higher (lower) risk of adverse clinical outcomes compared to that of baricitinib.

**FIGURE 1:**
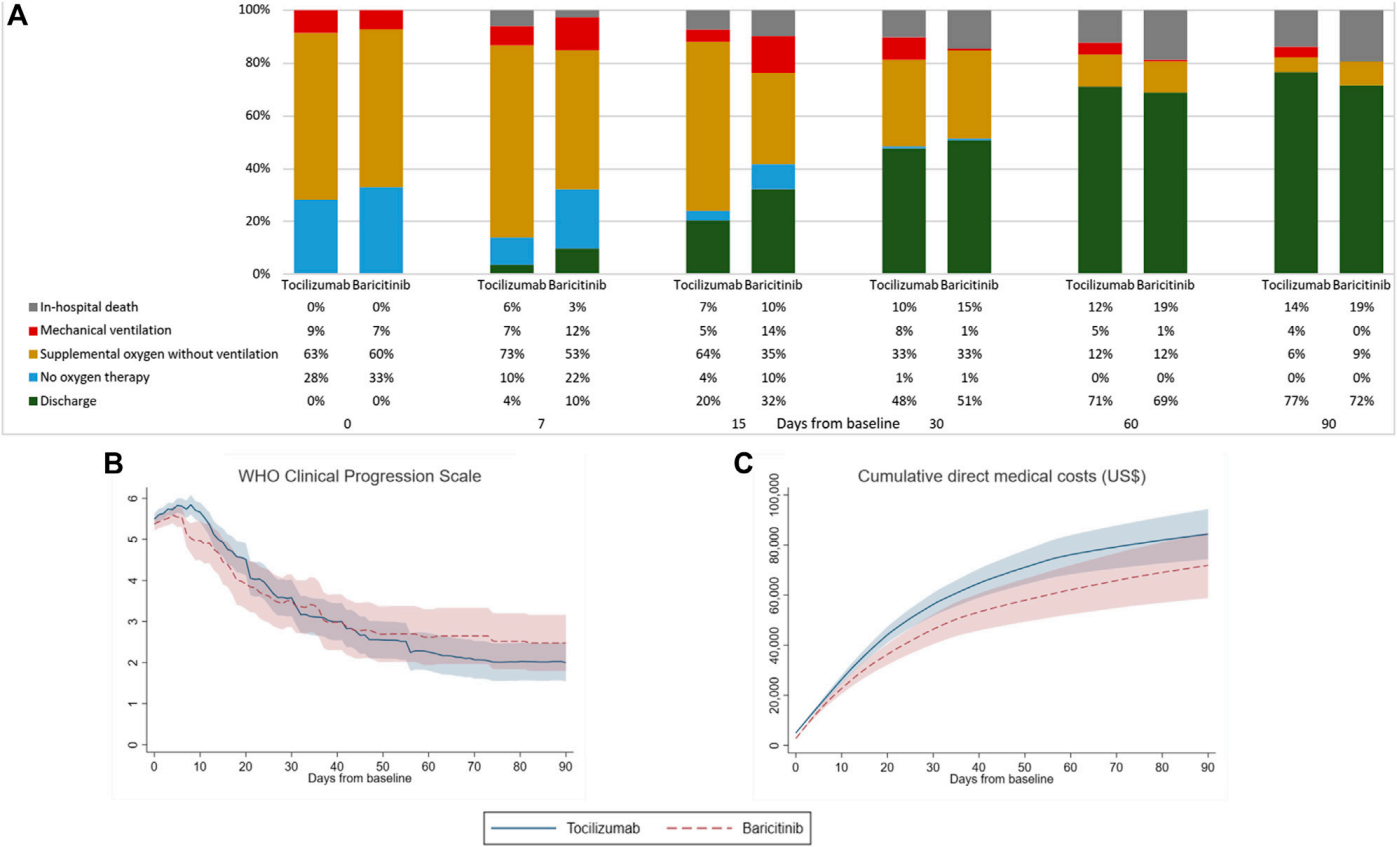
Comparison of **(A)** clinical status measured by WHO Clinical Progression Scale score, **(B)** WHO Clinical Progression Scale score, and **(C)** cumulative direct medical costs incurred by patients of tocilizumab and baricitinib groups from baseline to day-90 of follow-up.

Clinical status of patients from baseline to day-90 is illustrated in Figure 1 by treatment groups. The proportion of patients experiencing in-hospital death was numerically lower in the baricitinib group on day-7 versus that of tocilizumab (3 *vs.* 6%), which was reversed by day-90 (19 *vs*. 14%). Likewise, the percentage of patients discharged was initially higher among baricitinib users than their counterparts on tocilizumab (day-7: 10 *vs*. 4%), which was again reversed by day-90 (72 *vs*. 77%). Overall, the mean (standard deviation) hospital LOS of tocilizumab (43.9 [40.8] days) and baricitinib (40.3 [31.4] days) users were not significantly different (difference = 3.47 days, 95%CI -6.88 to 13.82, *p* = 0.509). Notably, the cumulative direct medical costs incurred were higher among tocilizumab users, albeit not significantly different from the baricitinib group since day-3 of follow-up.

**FIGURE 2:**
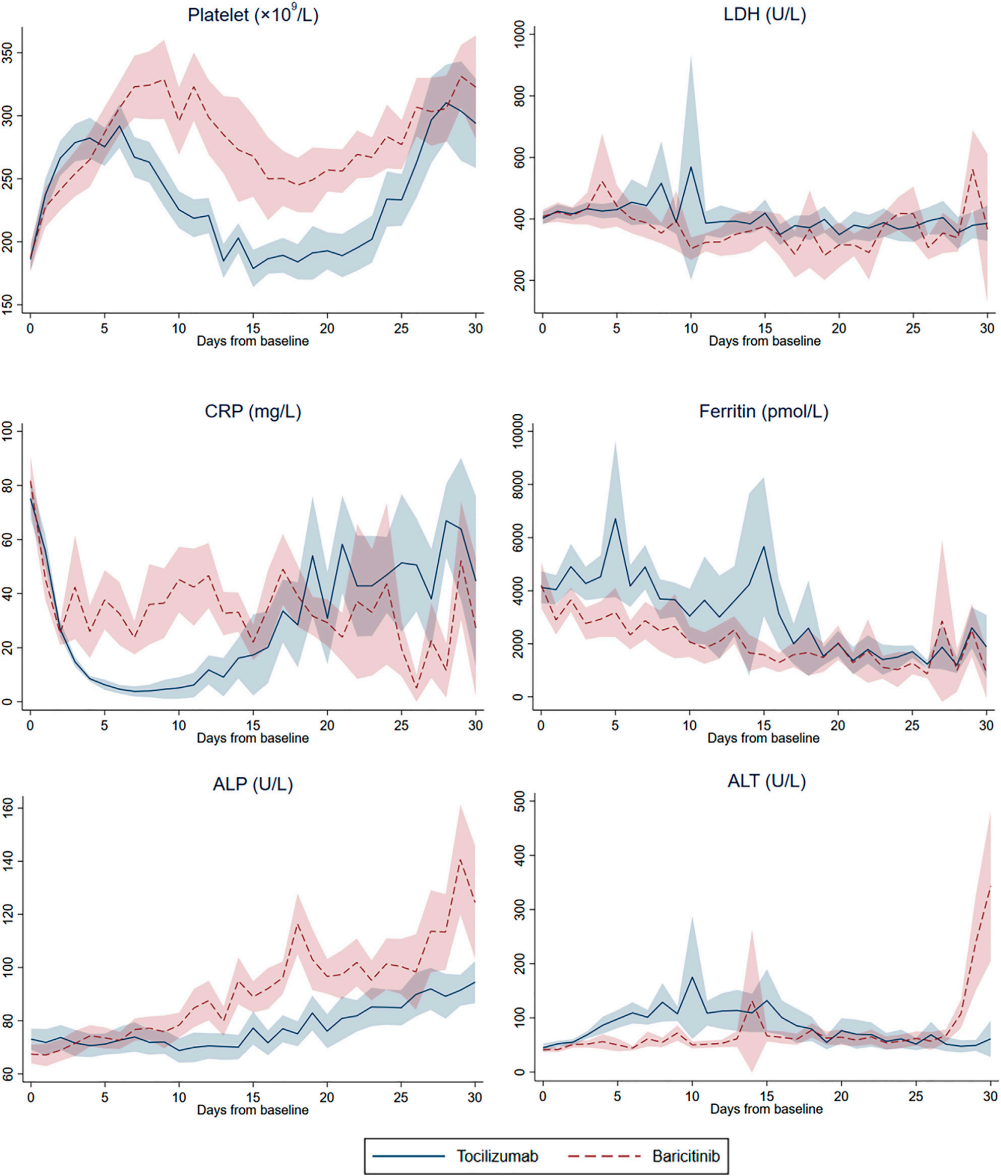
Daily mean (and 95% confidence interval) platelet count, lactate dehydrogenase (LDH), C-reactive protein (CRP), ferritin, alkaline phosphatase (ALP), and alanine aminotransferase (ALT) from baseline to day-30 of follow-up in tocilizumab and baricitinib groups.

As shown in Figure 2, the levels of various laboratory parameters fluctuated in both treatment groups over 30 days of follow-up. At baseline, patients from both treatment groups presented with similar abnormalities as follows: elevated neutrophil count, LDH, CK, CRP, and ferritin; lymphocyte count and hemoglobin level under the normal range; and Ct value < 35 cycles (Supplementary Table S6). Overall, significant changes could be observed from baseline to the last measurement across most laboratory parameters within each treatment group; yet paired differences between the two groups were generally comparable, except for a significantly larger decrease in hemoglobin among tocilizumab versus baricitinib users (−1.8 *vs*. −0.9 g/dl, *p* = 0.028).

Comparing to baseline observations, normalization of values was identified in lymphocyte count and CK level for both treatment groups at last measurements; while those of neutrophil count, LDH, CRP and ferritin remained elevated, and hemoglobin deviated further from the lower reference value. Despite both CRP and ferritin levels remained elevated at last measurements, tocilizumab and baricitinib use had resulted in comparable reduction in these inflammatory markers over the follow-up period, where all of these within-group differences were statistically significant. Remarkably, mean ALT levels of both treatment groups were above the upper reference value for the last measurement, with a numerically larger increase observed among tocilizumab (64.7 U/L, *p* = 0.068) versus baricitinib (27.5 U/L, *p* = 0.185) users (*p*-value for paired differences between groups = 0.362).

## Discussion

In this territory-wide cohort of patients admitted with moderate-to-severe COVID-19 and evidence of systemic inflammation, who were receiving dexamethasone during hospitalization, addition of tocilizumab or baricitinib had generally comparable effects on time to clinical improvement of at least one score reduction on the WHO CPS, hospital discharge, recovery without the need for oxygen therapy, low viral load, and positive IgG antibody, as well as hospital LOS. Tocilizumab users had a shorter time to viral clearance of marginal significance compared to those on baricitinib. Meanwhile, there were no significant differences in the risks of clinical deterioration, in-hospital death, severe liver injury, ARF, hyperinflammatory syndrome, thrombotic and bleeding events, and secondary infection between tocilizumab and baricitinib users.

Most studies comparing tocilizumab to control or standard care have reported no significant differences in time to clinical improvement (Salama et al., 2020; Stone et al., 2020; Hill et al., 2021; Rosas et al., 2021), with only one study reporting beneficial effects when tocilizumab was administered within 24 h of initiating organ support in the ICU among critically ill COVID-19 patients (The Remap-Cap Investigators, 2021); yet tocilizumab use was associated with shorter time to hospital discharge or higher discharge rates at day-28 (Hermine et al., 2021; RECOVERY Collaborative Group, 2021; Rosas et al., 2021; The REMAP-CAP Investigators, 2021). Meanwhile, baricitinib-remdesivir could promote faster clinical improvement compared to remdesivir alone in the ACTT-2 trial (Kalil et al., 2021). In view of the active comparator approach of our analysis, clinical outcomes associated with tocilizumab or baricitinib initiation were comparable for this patient cohort presented primarily with WHO CPS scores 5-6 (i.e. who required supplemental oxygen but not mechanical ventilation or ECMO) and evidence of hyperinflammatory syndrome at baseline. Our results were consistent with a retrospective cohort study (in preprint) that there were no significant differences between tocilizumab and baricitinib use with respect to mortality, improvement in respiratory status, and the risk of secondary infection in COVID-19 patients (Kojima et al., 2021), providing support to the current guidelines that either baricitinib or tocilizumab can be added to these patients on top of dexamethasone (Infectious Diseases Society of America, 2022; National Institutes of Health, 2022). With respect to viral dynamics, no significant differences in time to low viral load or positive IgG antibody were identified between treatment groups, which might be attributed to their comparable initial viral load, and antibody response was not impaired by either drug (Bronte et al., 2020; Masiá et al., 2020). While tocilizumab use was associated with a shorter time to viral clearance of marginal significance, further studies directly comparing tocilizumab and baricitinib in COVID-19 patients are needed to delineate their effects on SARS-CoV-2 RNA shedding, as the current literature is limited with inconclusive evidence on the association between the use of these immunomodulatory drugs and viral clearance, which is further complicated by the diverse clinical characteristics of COVID-19 patients (including but not limited to their age, albumin level, initial viral load, disease severity, and immunocompetence) (Masiá et al., 2020; Akbarzadeh-Khiavi et al., 2021; Cogliati Dezza et al., 2021).

For hospitalized patients with moderate-to-severe COVID-19, reducing the risks of clinical deterioration and mortality are of paramount importance. In the current analysis, the two treatment modalities were not significantly different in terms of in-hospital death and the composite outcome of disease progression. Compared to placebo or standard care, neither tocilizumab nor baricitinib has been shown to increase the risks of these clinical endpoints; whereas some studies have even demonstrated significantly better outcomes with their use, respectively (Guaraldi et al., 2020; Salama et al., 2020; Chen et al., 2021b; Kalil et al., 2021; Klopfenstein et al., 2021; Marconi et al., 2021; Pérez-Alba et al., 2021; RECOVERY Collaborative Group, 2021; Soin et al., 2021; Stebbing et al., 2021; The W. H. O. Rapid Evidence Appraisal for Covid-Therapies Working Group, 2021). A few observational studies have suggested that a significant reduction in mortality risk with tocilizumab use may be related to elevated CRP levels at baseline (e.g. >150 mg/L), or following a substantial decline in CRP with the drug (Biran et al., 2020; Canziani et al., 2020; Martínez-Sanz et al., 2021). Significant decreases in CRP were similarly achieved with either tocilizumab or baricitinib use in this patient cohort. Interestingly, an observational study has found that the use of tocilizumab outside ICU was associated with a sudden need of intubation among patients with severe COVID-19; however, such clinical deterioration was deemed temporary, where the respiratory function returned to normal or improved within a week (Rossotti et al., 2020). This might have translated to our comparable findings on disease progression and in-hospital death.

As dexamethasone, tocilizumab and baricitinib are all immunosuppressants, concerns about any additional risk of secondary infection have been raised, especially when a combination of these drugs is introduced (World Health Organization, 2021; Infectious Diseases Society of America, 2022; National Institutes of Health, 2022). The current literature has identified no significant differences or potentially an increased risk of new infections with tocilizumab (Guaraldi et al., 2020; Salama et al., 2020; Chen et al., 2021a; The W. H. O. Rapid Evidence Appraisal for Covid-Therapies Working Group, 2021), in contrast to baricitinib use associated with a possibly lower risk or an infection rate comparable to that of control (Kalil et al., 2021; Marconi et al., 2021; Pérez-Alba et al., 2021). With tocilizumab use, infection risk was particularly elevated among COVID-19 patients requiring invasive ventilation or intensive care (Hill et al., 2021; Somers et al., 2021). In this study cohort of patients with moderate-to-severe COVID-19, the risk of secondary infection was not significantly different between tocilizumab and baricitinib users; yet this finding should be interpreted with caution given the wide CI, potentially related to the small number of events.

Regarding the risks of COVID-19 complications, no significant differences were identified between tocilizumab and baricitinib groups in terms of severe liver injury, ARF, hyperinflammatory syndrome, and thrombotic and bleeding events. Both treatment modalities in our study managed to reduce the systemic inflammation of COVID-19 patients, as reflected by their substantial decreases in CRP and ferritin levels. While numerically larger increases in LDH and ALT levels were identified with tocilizumab use, paired differences of these measurements between the two treatment groups were not significant, hence their comparable risk on severe liver injury. Both tocilizumab and baricitinib use was associated with significant within-group increases in platelet count over the follow-up period, whereas paired differences between treatment groups were comparable; and notably, both groups had mean platelet count within the normal range for both baseline and last follow-up measurements. Our results suggest that baricitinib use in patients hospitalized with COVID-19 was unlikely to pose an additional risk of thrombotic and bleeding events compared to that of tocilizumab, which is in line with current evidence that no clear temporal or quantitative association has been established between increases in platelet count and thromboembolic events (Jorgensen et al., 2020). Similar changes in these laboratory parameters have been illustrated in previous studies of tocilizumab and baricitinib (Jorgensen et al., 2020; Rossotti et al., 2020; Atzeni et al., 2021; Corominas et al., 2021).

Based on a territory-wide cohort of patients hospitalized with COVID-19, our study has offered the much-needed evidence on a head-to-head comparison between tocilizumab and baricitinib, establishing their safety and efficacy on top of background dexamethasone use. Our study cohort has included all eligible patients from the population hospitalized with COVID-19 in the local region, comprising cases over the spectrum of disease severity. Meanwhile, our results could be influenced by several limitations of this study. Firstly, owing to its retrospective nature, unmeasured or residual confounding could remain. Besides, selection or indication biases on the prescription of specific drugs might not have been fully eliminated, despite our statistical approach of IPTW using PS to account for differences in baseline covariates between treatment groups. The use of deidentified, anonymous patient data in this retrospective cohort study also prohibited the determination of clinical criteria for which individual patients were prescribed with specific drugs. Secondly, our results might not be generalizable to COVID-19 patients whose characteristics were different from our cohort at drug initiation, such as disease severity and the use of concomitant treatments (majority of our patients were on interferon-β-1b). Furthermore, our patient population was relatively small with heterogenous disease severity, hence further studies with larger sample sizes are needed to confirm our results of the head-to-head comparison between tocilizumab and baricitinib. Thirdly, IL-6 measurement was not routinely taken in local practices, hence relevant data were not available for the evaluation of tocilizumab use. Lastly, study outcomes could be affected by the progression of COVID-19 itself, rather than solely the effects of respective drug regimens.

Among hospitalized patients with moderate-to-severe COVID-19, our study has observed that on top of dexamethasone, the addition of tocilizumab or baricitinib had generally comparable effects on time to clinical improvement of at least one score reduction on the WHO CPS, hospital discharge, recovery without the need for oxygen therapy, low viral load, and positive IgG antibody, as well as hospital LOS. Tocilizumab users had a shorter time to viral clearance of marginal significance compared to those on baricitinib. Meanwhile, the initiation of tocilizumab was associated with comparable risks of clinical deterioration, in-hospital death, sever liver injury, ARF, hyperinflammatory syndrome, thrombotic and bleeding events, and secondary infection to that of baricitinib. Given our relatively small sample size with heterogenous disease severity, further studies and clinical trials are needed to confirm our findings regarding the evaluation of tocilizumab and baricitinib use among COVID-19 patients of different disease severities, at varying stages or timing of drug initiation, and considering the concomitant use of other therapeutics. Such clinical evidence will be essential to informing and reviewing the recommendations of treatment options for managing patients with COVID-19.

## Data Availability

The data that support the findings of this study were provided by the Hong Kong Hospital Authority. Restrictions apply to the availability of these data, which were used under license for this study. Requests to access these datasets should be directed to the Hospital Authority, hacpaaedr@ha.org.hk.
