# Supplementary material for: Initiation of Tocilizumab or Baricitinib Were Associated With Comparable Clinical Outcomes Among Patients Hospitalized With COVID-19 and Treated With Dexamethasone
Source: Front Pharmacol. 2022 May 30;13:866441. doi: 10.3389/fphar.2022.866441 (PMC9189358; doi:10.3389/fphar.2022.866441)
Supplement: Supplementary file 1 [file DataSheet1.docx]

Supplementary Material

# Supplementary Figures

Supplementary Figure 1.

(a) Distribution of timing of tocilizumab and baricitinib initiation by two groups


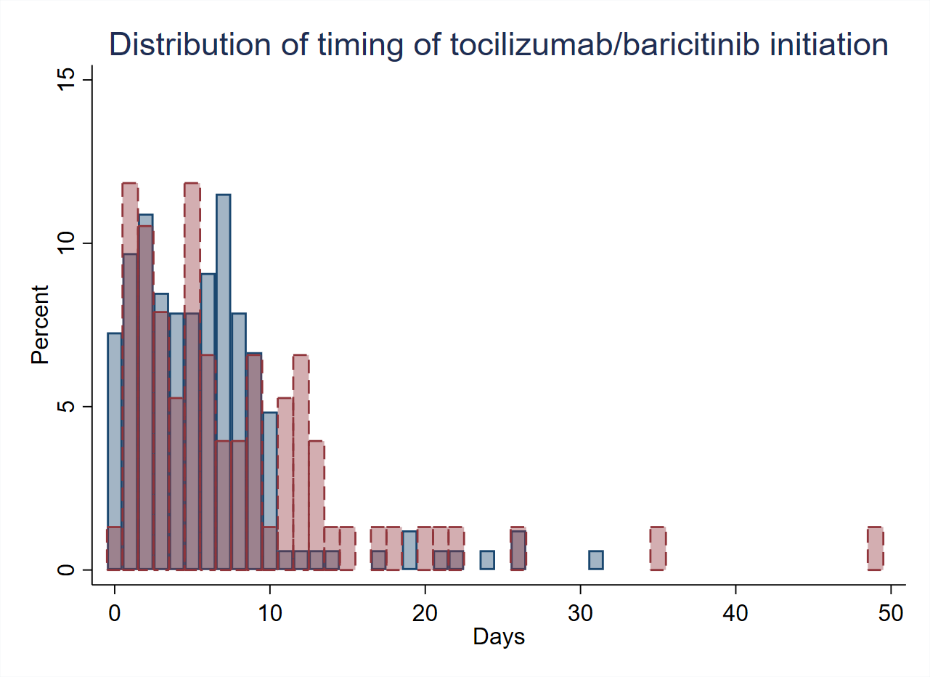
(b) Distribution of timing of dexamethasone initiation by two groups


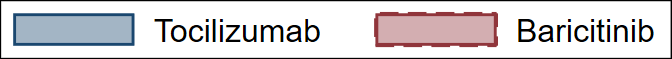

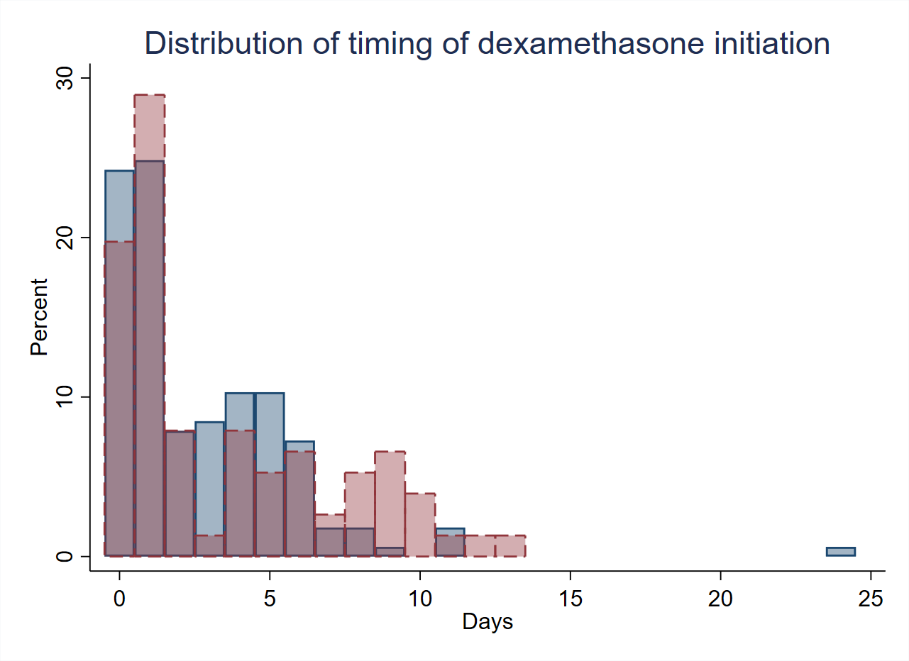


Supplementary Figure 2. Flowchart of inclusion and exclusion of patients hospitalized with COVID-19 who had initiated tocilizumab or baricitinib on top of dexamethasone during hospitalization


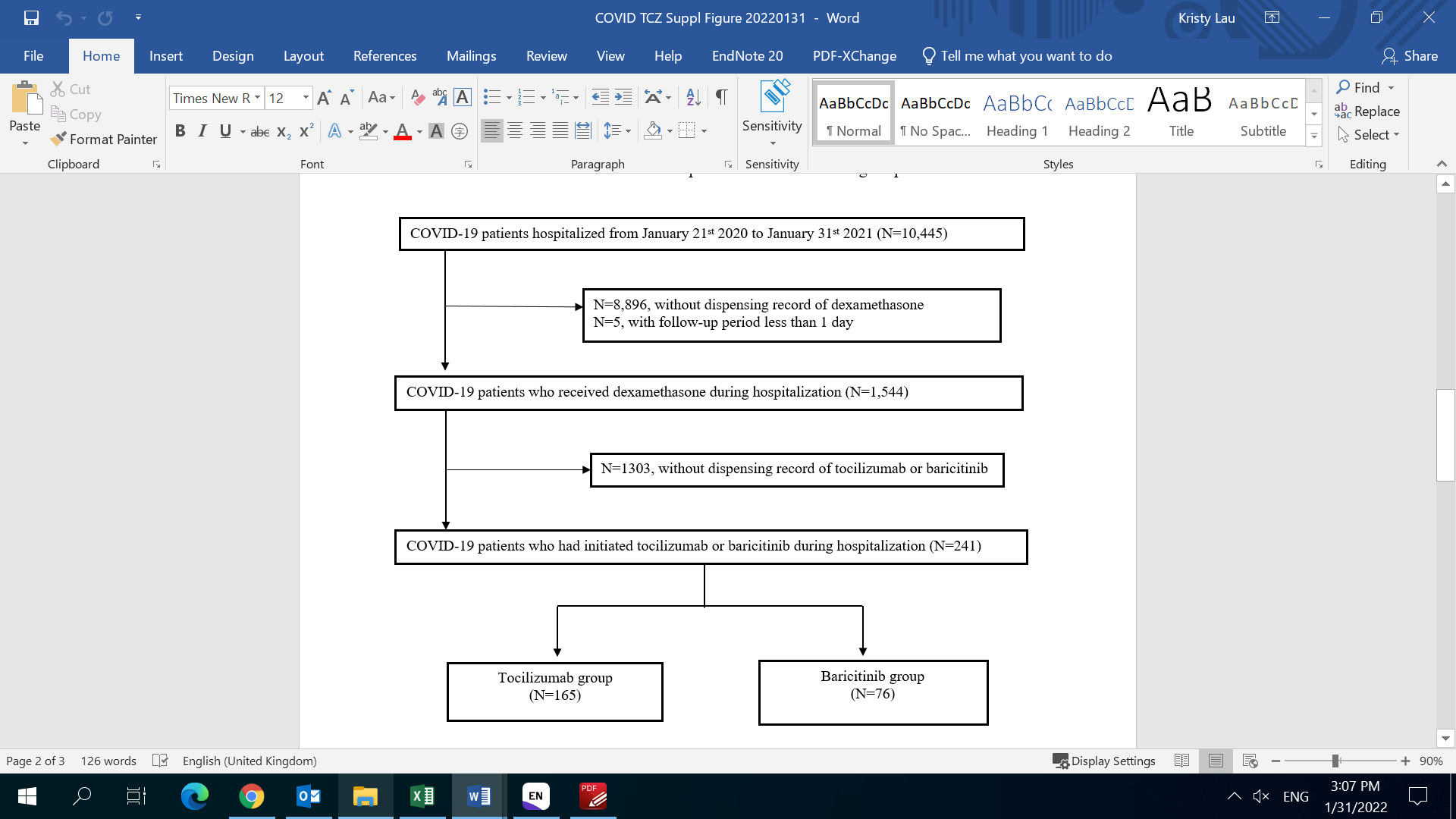


Supplementary Figure 3. Distribution of propensity score density by the two treatment groups before and after weighting


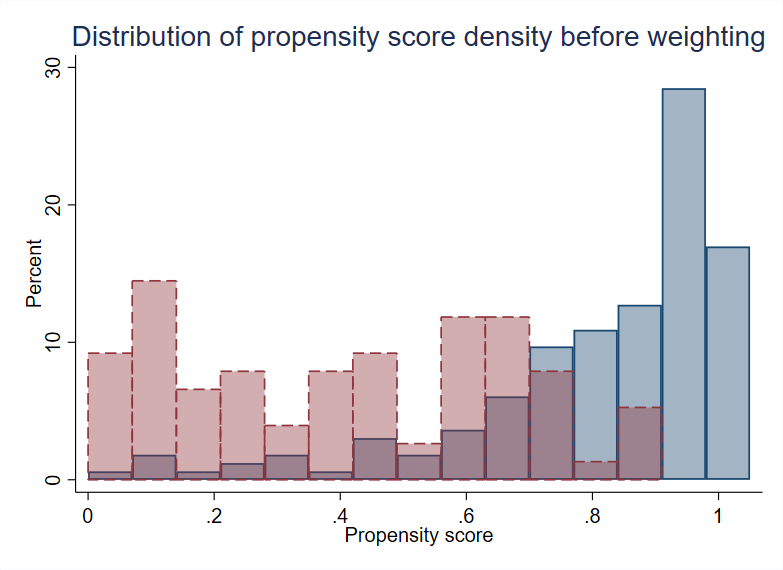


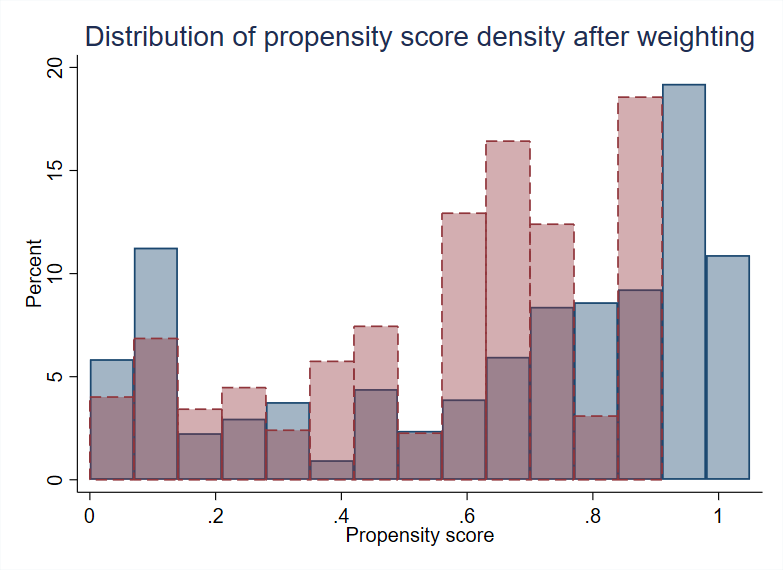

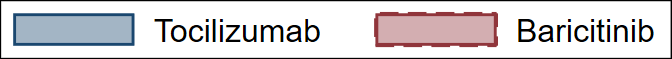


# Supplementary Tables

Supplementary Table 1. Unit cost of relevant medical treatments and healthcare services

| Medication / Health Service | Cost (HK$) | Cost (US$)^†^ |
| --- | --- | --- |
| Tocilizumab 80 mg intravenous, per dose | 3,829 | 491 |
| Tocilizumab 200 mg intravenous, per dose | 9,462 | 1,213 |
| Tocilizumab 400 mg intravenous, per dose | 18,851 | 2,417 |
| Baricitinib 2mg oral, per day | 618 | 79 |
| Remdesivir, per vial | 3,042 | 390 |
| Dexamethasone, per 10 days | 94 | 12 |
| Hospitalization, per day |  |  |
| General Ward | 5,100 | 654 |
| Intensive Care Unit | 24,400 | 3,128 |
| Emergency department, per visit | 1,230 | 158 |
| Polymerase chain reaction tests | 240 | 31 |
| Extracorporeal membrane oxygenation (ECMO) catheter insertion | 52,100 | 6,679 |
| ECMO | 80,175 | 10,279 |
| Tracheostomy | 16,050 | 2,058 |
| Hemodialysis | 9,410 | 1,206 |
| Peritoneal dialysis | 9,410 | 1,206 |

Data source: Hong Kong SAR Government Gazette and Hospital Authority Ordinance (chapter 113): Public charges – non-eligible persons.

<https://www.gilead.com/news-and-press/press-room/press-releases/2020/6/an-open-letter-from-daniel-oday-chairman--ceo-gilead-sciences>

<https://www.drugs.com/price-guide/actemra>

<https://www.lillypricinginfo.com/olumiant>

Note:

†A fixed exchange rate of 1 US$=7.8 HK$

Supplementary Table 2. Data completion rates of patient characteristics at baseline before multiple imputation

| Characteristics | Total (N = 241) | Tocilizumab (N=165) | Baricitinib (N=76) |
| --- | --- | --- | --- |
| Age | 100.0% (241) | 100.0% (165) | 100.0% (76) |
| Sex | 100.0% (241) | 100.0% (165) | 100.0% (76) |
| Time of treatment initiation | 100.0% (241) | 100.0% (165) | 100.0% (76) |
| Pre-existing comorbidities on admission |  |  |  |
| Charlson Comorbidity Index | 100.0% (241) | 100.0% (165) | 100.0% (76) |
| Diabetes mellitus | 100.0% (241) | 100.0% (165) | 100.0% (76) |
| Hypertension | 100.0% (241) | 100.0% (165) | 100.0% (76) |
| Liver disease | 100.0% (241) | 100.0% (165) | 100.0% (76) |
| Chronic lung disease | 100.0% (241) | 100.0% (165) | 100.0% (76) |
| Chronic heart disease | 100.0% (241) | 100.0% (165) | 100.0% (76) |
| Chronic kidney disease | 100.0% (241) | 100.0% (165) | 100.0% (76) |
| Long-term medications |  |  |  |
| Anticoagulant | 100.0% (241) | 100.0% (165) | 100.0% (76) |
| Antiplatelet | 100.0% (241) | 100.0% (165) | 100.0% (76) |
| Treatment performed prior to baseline |  |  |  |
| Tocilizumab | 100.0% (241) | 100.0% (165) | 100.0% (76) |
| Time from admission to tocilizumab initiation, days | 100.0% (165) | 100.0% (165) | NA |
| Duration of use of tocilizumab, days | 100.0% (165) | 100.0% (165) | NA |
| Cumulative dosage of tocilizumab, mg | 100.0% (165) | 100.0% (165) | NA |
| Baricitinib | 100.0% (241) | 100.0% (165) | 100.0% (76) |
| Time from admission to baricitinib initiation, days | 100.0% (68) | NA | 100.0% (76) |
| Duration of use of baricitinib, days | 100.0% (68) | NA | 100.0% (76) |
| Cumulative dosage of baricitinib, mg | 100.0% (68) | NA | 100.0% (76) |
| Dexamethasone | 100.0% (241) | 100.0% (165) | 100.0% (76) |
| Time from admission to dexamethasone initiation, days | 100.0% (241) | 100.0% (165) | 100.0% (76) |
| Duration of use of dexamethasone, days | 100.0% (241) | 100.0% (165) | 100.0% (76) |
| Cumulative dosage of dexamethasone, mg | 100.0% (241) | 100.0% (165) | 100.0% (76) |
| Administration route of dexamethasone | 100.0% (241) | 100.0% (165) | 100.0% (76) |
| Dosage of dexamethasone | 100.0% (241) | 100.0% (165) | 100.0% (76) |
| Remdesivir | 100.0% (241) | 100.0% (165) | 100.0% (76) |
| Time from admission to remdesivir initiation, days | 100.0% (68) | NA | 92.1% (70) |
| Duration of use of remdesivir, days | 100.0% (68) | NA | 92.1% (70) |
| Cumulative dosage of remdesivir, mg | 100.0% (68) | NA | 92.1% (70) |
| Interferon-β-1b | 100.0% (241) | 100.0% (165) | 100.0% (76) |
| Ribavirin | 100.0% (241) | 100.0% (165) | 100.0% (76) |
| Other systemic steroid | 100.0% (241) | 100.0% (165) | 100.0% (76) |
| ECMO | 100.0% (241) | 100.0% (165) | 100.0% (76) |
| Invasive mechanical ventilation | 100.0% (241) | 100.0% (165) | 100.0% (76) |
| Dialysis | 100.0% (241) | 100.0% (165) | 100.0% (76) |
| ICU admission | 100.0% (241) | 100.0% (165) | 100.0% (76) |
| Clinical severity by WHO Clinical Progression Scale | 100.0% (241) | 100.0% (165) | 100.0% (76) |
| Severe liver injury | 100.0% (241) | 100.0% (165) | 100.0% (76) |
| Acute renal failure | 100.0% (241) | 100.0% (165) | 100.0% (76) |
| Hepatic inflammation | 100.0% (241) | 100.0% (165) | 100.0% (76) |
| Hyperinflammatory syndrome | 100.0% (241) | 100.0% (165) | 100.0% (76) |
| Thrombotic and bleeding events | 100.0% (241) | 100.0% (165) | 100.0% (76) |
| Laboratory parameters |  |  |  |
| White blood cell | 99.2% (239) | 100.0% (165) | 97.4% (74) |
| Neutrophil | 99.2% (239) | 100.0% (165) | 97.4% (74) |
| Lymphocyte | 99.2% (239) | 100.0% (165) | 97.4% (74) |
| Platelet | 99.2% (239) | 100.0% (165) | 97.4% (74) |
| Lactate dehydrogenase | 97.9% (236) | 100.0% (165) | 93.4% (71) |
| Creatine kinase | 89.2% (215) | 90.9% (150) | 85.5% (65) |
| Total bilirubin | 99.6% (240) | 100.0% (165) | 98.7% (75) |
| C-reactive protein | 100.0% (241) | 100.0% (165) | 100.0% (76) |
| Ferritin | 85.5% (206) | 87.9% (145) | 80.3% (61) |
| Cycle threshold value | 95.9% (231) | 97.6% (161) | 92.1% (70) |
| eGFR | 100.0% (241) | 100.0% (165) | 100.0% (76) |
| ALP | 99.6% (240) | 100.0% (165) | 98.7% (75) |
| ALT | 99.6% (240) | 100.0% (165) | 98.7% (75) |
| Hemoglobin | 99.2% (239) | 100.0% (165) | 97.4% (74) |
|  |  |  |  |

Note:

ALP = alkaline phosphatase; ALT = alanine aminotransferase; ECMO = extracorporeal membrane oxygenation; eGFR = estimated glomerular filtration rate; ICU = intensive care unit; SOFA = Sequential Organ Failure Assessment

Supplementary Table 3. Number and incidence rate of outcome events in tocilizumab and baricitinib groups before and after weighting

|  | Before weighting | | | | | | |  | After weighting | |
| --- | --- | --- | --- | --- | --- | --- | --- | --- | --- | --- |
| Event | Cumulative incidence | | Crude incidence rate  (Events / 1,000 person-days) | | | Median follow-up periods (Days) | Mean follow-up periods (Days) |  | Crude incidence rate  (Events / 1,000 person-days) | |
|  | Cases with event | Rate | Estimate | 95% CI* | Person-days |  |  |  | Estimate | 95% CI* |
| **Total (N = 241)** |  |  |  |  |  |  |  |  |  |  |
| Clinical improvement on WHO clinical progression scale by ≥ 1 score | 202 | 83.8% | 28.29 | (24.52, 32.47) | 7,141 | 18 | 30 |  | 27.95 | (25.16, 30.90) |
| Hospital discharge (score ≤ 3) | 193 | 80.1% | 21.03 | (18.16, 24.21) | 9,179 | 21 | 38 |  | 22.83 | (20.53, 25.29) |
| Recovery (score ≤ 4) | 124 | 73.4% | 15.72 | (13.08, 18.74) | 7,888 | 27 | 47 |  | 17.69 | (15.46, 20.07) |
| Viral clearance (first negative PCR result) | 98 | 43.2% | 20.48 | (16.62, 24.95) | 4,786 | 16 | 21 |  | 17.11 | (14.59, 19.91) |
| Low viral load (Ct value ≥ 35) | 98 | 43.2% | 19.39 | (15.74, 23.63) | 5,054 | 15 | 22 |  | 22.84 | (19.75, 26.25) |
| IgG antibody | 163 | 85.3% | 84.50 | (72.03, 98.51) | 1,929 | 8 | 10 |  | 81.49 | (72.33, 91.26) |
| In-hospital death or invasive mechanical ventilation (score ≥ 7) or intensive care unit admission | 64 | 31.5% | 14.93 | (11.49, 19.06) | 4,288 | 16 | 21 |  | 14.95 | (12.34, 17.87) |
| In-hospital death (score = 10) | 44 | 18.3% | 4.79 | (3.48, 6.44) | 9,179 | 21 | 38 |  | 4.43 | (3.43, 5.57) |
| Severe liver injury | 40 | 16.8% | 5.58 | (3.98, 7.59) | 7,172 | 18 | 30 |  | 4.90 | (3.76, 6.28) |
| Acute renal failure | 23 | 9.6% | 2.69 | (1.71, 4.04) | 8,540 | 21 | 36 |  | 2.92 | (2.10, 3.88) |
| Hyperinflammatory syndrome | 25 | 78.1% | 116.82 | (75.60, 172.45) | 214 | 2 | 7 |  | 148.96 | (109.70, 194.28) |
| Secondary infection | 14 | 5.9% | 1.80 | (0.98, 3.01) | 7,796 | 21 | 33 |  | 1.17 | (0.67, 1.86) |
| Thrombotic and bleeding events | 17 | 9.3% | 3.70 | (2.16, 5.93) | 4,592 | 15 | 25 |  | 3.76 | (2.41, 5.54) |
| **Tocilizumab (N = 165)** |  |  |  |  |  |  |  |  |  |  |
| Clinical improvement on WHO clinical progression scale by ≥ 1 score | 140 | 84.8% | 27.12 | (22.81, 32.00) | 5,162 | 18 | 31 |  | 26.69 | (23.29, 30.35) |
| Hospital discharge (score ≤ 3) | 133 | 80.6% | 20.03 | (16.77, 23.74) | 6,640 | 21 | 40 |  | 21.47 | (18.71, 24.49) |
| Recovery (score ≤ 4) | 89 | 75.4% | 15.47 | (12.42, 19.03) | 5,754 | 26 | 49 |  | 17.80 | (15.06, 20.86) |
| Viral clearance (first negative PCR result) | 74 | 47.7% | 25.35 | (19.91, 31.83) | 2,919 | 15 | 19 |  | 23.56 | (19.49, 28.06) |
| Low viral load (Ct value ≥ 35) | 73 | 46.8% | 19.65 | (15.40, 24.71) | 3,715 | 13 | 24 |  | 24.11 | (20.25, 28.49) |
| IgG antibody | 118 | 85.5% | 85.01 | (70.37, 101.81) | 1,388 | 8 | 10 |  | 84.26 | (72.55, 97.01) |
| In-hospital death or invasive mechanical ventilation (score ≥ 7) or intensive care unit admission | 46 | 33.6% | 15.91 | (11.65, 21.22) | 2,891 | 16 | 21 |  | 14.11 | (10.99, 17.84) |
| In-hospital death (score = 10) | 29 | 17.6% | 4.37 | (2.92, 6.27) | 6,640 | 21 | 40 |  | 3.48 | (2.42, 4.80) |
| Severe liver injury | 30 | 18.5% | 5.93 | (4.00, 8.46) | 5,063 | 18 | 31 |  | 4.83 | (3.41, 6.52) |
| Acute renal failure | 19 | 11.6% | 3.17 | (1.91, 4.94) | 6,001 | 21 | 37 |  | 3.73 | (2.59, 5.14) |
| Hyperinflammatory syndrome | 16 | 88.9% | 320.00 | (182.91, 519.66) | 50 | 1 | 3 |  | 386.51 | (264.22, 539.06) |
| Secondary infection | 12 | 7.4% | 2.21 | (1.14, 3.87) | 5,418 | 21 | 33 |  | 1.56 | (0.82, 2.58) |
| Thrombotic and bleeding events | 15 | 10.6% | 3.81 | (2.13, 6.28) | 3,940 | 16 | 28 |  | 3.36 | (1.90, 5.32) |
| **Baricitinib (N = 76)** |  |  |  |  |  |  |  |  |  |  |
| Clinical improvement on WHO clinical progression scale by ≥ 1 score | 62 | 81.6% | 31.33 | (24.02, 40.16) | 1,979 | 19 | 26 |  | 30.15 | (25.46, 35.42) |
| Hospital discharge (score ≤ 3) | 60 | 78.9% | 23.63 | (18.03, 30.42) | 2,539 | 22 | 33 |  | 25.29 | (21.23, 29.71) |
| Recovery (score ≤ 4) | 35 | 68.6% | 16.40 | (11.42, 22.81) | 2,134 | 31 | 42 |  | 17.50 | (13.89, 21.58) |
| Viral clearance (first negative PCR result) | 24 | 33.3% | 12.85 | (8.24, 19.13) | 1,867 | 17 | 26 |  | 9.78 | (7.06, 13.05) |
| Low viral load (Ct value ≥ 35) | 25 | 35.2% | 18.67 | (12.08, 27.56) | 1,339 | 16 | 19 |  | 20.37 | (15.50, 26.20) |
| IgG antibody | 45 | 84.9% | 83.18 | (60.67, 111.30) | 541 | 8 | 10 |  | 76.89 | (62.79, 93.12) |
| In-hospital death or invasive mechanical ventilation (score ≥ 7) or intensive care unit admission | 18 | 27.3% | 12.88 | (7.64, 20.36) | 1,397 | 17 | 21 |  | 16.35 | (12.00, 21.59) |
| In-hospital death (score = 10) | 15 | 19.7% | 5.91 | (3.31, 9.74) | 2,539 | 22 | 33 |  | 6.14 | (4.25, 8.54) |
| Severe liver injury | 10 | 13.2% | 4.74 | (2.27, 8.72) | 2,109 | 20 | 28 |  | 5.04 | (3.14, 7.58) |
| Acute renal failure | 4 | 5.3% | 1.58 | (0.43, 4.03) | 2,539 | 22 | 33 |  | 1.54 | (0.67, 2.92) |
| Hyperinflammatory syndrome | 9 | 64.3% | 54.88 | (25.09, 104.18) | 164 | 9 | 12 |  | 69.27 | (40.13, 108.62) |
| Secondary infection | 2 | 2.6% | 0.84 | (0.10, 3.04) | 2,378 | 21 | 31 |  | 0.53 | (0.08, 1.48) |
| Thrombotic and bleeding events | 2 | 4.9% | 3.07 | (0.37, 11.08) | 652 | 15 | 16 |  | 5.05 | (2.02, 9.74) |

Note:

CI = confidence interval; Ct = cycle threshold; IgG = immunoglobulin G; PCR = polymerase chain reaction

* 95% CI of incidence rates were constructed by Poisson distribution

Supplementary Table 4. Sensitivity analyses on study outcomes

|  | Before weighting | | After weighting | | |
| --- | --- | --- | --- | --- | --- |
|  | Tocilizumab | Baricitinib | Tocilizumab vs Baricitinib | | |
|  | % (N) | % (N) | HR† | 95% CI | P-value |
| **Clinical improvement on WHO clinical progression scale by ≥ 1 score** | | | | | |
| Removing hospital discharge as censoring criteria | 83.0% (165) | 77.6% (76) | 1.00 | (0.70, 1.45) | 0.979 |
| At most 90 days of follow-up period | 75.8% (165) | 76.3% (76) | 0.91 | (0.58, 1.44) | 0.694 |
| Perform complete-case using the IPTW | 83.8% (148) | 83.6% (61) | 0.83 | (0.60, 1.15) | 0.269 |
| **Hospital discharge (score ≤ 3)** |  |  |  |  |  |
| Removing hospital discharge as censoring criteria | 79.4% (165) | 75.0% (76) | 0.98 | (0.67, 1.43) | 0.910 |
| At most 90 days of follow-up period | 71.5% (165) | 73.7% (76) | 0.86 | (0.54, 1.37) | 0.524 |
| Perform complete-case using the IPTW | 79.1% (148) | 80.3% (61) | 0.83 | (0.58, 1.20) | 0.327 |
| **Recovery (score ≤ 4)** |  |  |  |  |  |
| Removing hospital discharge as censoring criteria | 75.4% (118) | 68.6% (51) | 1.15 | (0.72, 1.83) | 0.554 |
| At most 90 days of follow-up period | 64.4% (118) | 66.7% (51) | 1.03 | (0.58, 1.85) | 0.913 |
| Perform complete-case using the IPTW | 74.3% (109) | 69.8% (43) | 0.82 | (0.52, 1.29) | 0.392 |
| **Viral clearance (first negative PCR result)** |  |  |  |  |  |
| Removing hospital discharge as censoring criteria | 47.7% (155) | 33.3% (72) | 2.20 | (1.20, 4.01) | 0.011 |
| At most 90 days of follow-up period | 47.7% (155) | 33.3% (72) | 1.94 | (1.01, 3.73) | 0.048 |
| Perform complete-case using the IPTW | 45.3% (139) | 34.5% (58) | 1.53 | (0.82, 2.87) | 0.179 |
| **Low viral load (Ct value ≥ 35)** |  |  |  |  |  |
| Removing hospital discharge as censoring criteria | 46.8% (156) | 35.2% (71) | 1.77 | (0.99, 3.15) | 0.053 |
| At most 90 days of follow-up period | 46.2% (156) | 35.2% (71) | 1.49 | (0.85, 2.60) | 0.162 |
| Perform complete-case using the IPTW | 47.1% (140) | 36.2% (58) | 1.64 | (0.93, 2.90) | 0.085 |
| **IgG antibody** |  |  |  |  |  |
| Removing hospital discharge as censoring criteria | 85.5% (138) | 84.9% (53) | 0.99 | (0.63, 1.58) | 0.977 |
| At most 90 days of follow-up period | 85.5% (138) | 84.9% (53) | 0.97 | (0.61, 1.54) | 0.909 |
| Perform complete-case using the IPTW | 86.1% (122) | 81.0% (42) | 1.05 | (0.67, 1.63) | 0.833 |
| **In-hospital death or invasive mechanical ventilation (score ≥ 7) or intensive care unit admission** | | | | | |
| Removing hospital discharge as censoring criteria | 33.6% (137) | 25.8% (66) | 1.10 | (0.51, 2.37) | 0.802 |
| At most 90 days of follow-up period | 32.8% (137) | 25.8% (66) | 0.98 | (0.46, 2.06) | 0.949 |
| Perform complete-case using the IPTW | 37.5% (120) | 28.3% (53) | 1.60 | (0.81, 3.14) | 0.172 |
| **In-hospital death (score = 10)** |  |  |  |  |  |
| Removing hospital discharge as censoring criteria | 17.6% (165) | 19.7% (76) | 0.70 | (0.33, 1.48) | 0.351 |
| At most 90 days of follow-up period | 17.6% (165) | 19.7% (76) | 0.63 | (0.29, 1.35) | 0.233 |
| Perform complete-case using the IPTW | 18.9% (148) | 18.0% (61) | 0.73 | (0.36, 1.50) | 0.396 |
| **Severe liver injury** |  |  |  |  |  |
| Removing hospital discharge as censoring criteria | 18.5% (162) | 13.2% (76) | 1.29 | (0.47, 3.51) | 0.621 |
| At most 90 days of follow-up period | 17.9% (162) | 13.2% (76) | 1.11 | (0.41, 2.97) | 0.834 |
| Perform complete-case using the IPTW | 19.3% (145) | 11.5% (61) | 1.99 | (0.82, 4.85) | 0.129 |
| **Acute renal failure** |  |  |  |  |  |
| Removing hospital discharge as censoring criteria | 11.6% (164) | 3.9% (76) | 3.38 | (0.78, 14.56) | 0.102 |
| At most 90 days of follow-up period | 10.4% (164) | 3.9% (76) | 2.54 | (0.59, 10.88) | 0.210 |
| Perform complete-case using the IPTW | 12.9% (147) | 4.9% (61) | 1.36 | (0.36, 5.16) | 0.653 |
| **Hyperinflammatory syndrome** |  |  |  |  |  |
| Removing hospital discharge as censoring criteria | 88.9% (18) | 64.3% (14) | 2.16 | (0.83, 5.62) | 0.112 |
| At most 90 days of follow-up period | 88.9% (18) | 64.3% (14) | 2.32 | (0.87, 6.25) | 0.091 |
| Perform complete-case using the IPTW | 94.1% (17) | 62.5% (8) | 3.76 | (0.99, 14.33) | 0.052 |
| **Secondary infection** |  |  |  |  |  |
| Removing hospital discharge as censoring criteria | 7.4% (163) | 2.6% (76) | 2.88 | (0.59, 13.99) | 0.189 |
| At most 90 days of follow-up period | 6.1% (163) | 2.6% (76) | 2.54 | (0.52, 12.48) | 0.250 |
| Perform complete-case using the IPTW | 8.2% (146) | 3.3% (61) | 2.80 | (0.56, 14.02) | 0.210 |
| **Thrombotic and bleeding events** |  |  |  |  |  |
| Removing hospital discharge as censoring criteria | 10.6% (141) | 4.9% (41) | 1.40 | (0.33, 6.06) | 0.648 |
| At most 90 days of follow-up period | 10.6% (141) | 4.9% (41) | 1.39 | (0.32, 6.00) | 0.658 |
| Perform complete-case using the IPTW | 12.1% (124) | 6.3% (32) | 2.10 | (0.43, 10.16) | 0.354 |
|  |  |  |  |  |  |

Note:

CI = confidence interval; Ct = cycle threshold; HR = hazard ratio; IgG = immunoglobulin G; IPTW = inverse probability of treatment weighting; PCR = polymerase chain reaction

† HR >1 (or <1) indicates that tocilizumab use was associated with better (worse) clinical improvement, earlier (later) hospital discharge or recovery, or higher (lower) risk of adverse clinical outcomes compared to that of baricitinib

Supplementary Table 5. Subgroup analyses on study outcomes

|  | Before weighting | | After weighting | | |
| --- | --- | --- | --- | --- | --- |
|  | Tocilizumab | Baricitinib | Tocilizumab vs Baricitinib | | |
|  | % (N) | % (N) | HR† | 95% CI | P-value |
| **Clinical improvement on WHO clinical progression scale by ≥ 1 score** | | | | | |
| Overall | 84.8% (165) | 81.6% (76) | 0.86 | (0.57, 1.29) | 0.459 |
| Age ≤ 65 | 97.2% (71) | 96.3% (27) | 0.72 | (0.45, 1.17) | 0.184 |
| Age > 65 | 75.5% (94) | 73.5% (49) | 0.83 | (0.49, 1.42) | 0.502 |
| Male | 86.8% (106) | 80.0% (45) | 1.01 | (0.59, 1.72) | 0.980 |
| Female | 81.4% (59) | 83.9% (31) | 0.61 | (0.37, 1.01) | 0.053 |
| Timing of dexamethasone initiation - within the first 3 days of admission | 87.0% (108) | 75.0% (44) | 0.72 | (0.46, 1.11) | 0.138 |
| Timing of dexamethasone initiation - within the first 5 days of admission | 87.3% (142) | 77.8% (54) | 0.74 | (0.50, 1.11) | 0.144 |
| Timing of dexamethasone initiation - within the first 7 days of admission | 86.6% (157) | 80.3% (61) | 0.87 | (0.55, 1.38) | 0.553 |
| Timing of tocilizumab initiation - within the first 3 days of admission | 88.3% (60) | 81.6% (76) | 0.73 | (0.44, 1.21) | 0.218 |
| Timing of tocilizumab initiation - within the first 5 days of admission | 86.0% (86) | 81.6% (76) | 0.89 | (0.57, 1.39) | 0.610 |
| Timing of tocilizumab initiation - within the first 7 days of admission | 87.5% (120) | 81.6% (76) | 0.87 | (0.57, 1.33) | 0.521 |
| Timing of baricitinib initiation - within the first 3 days of admission | 84.8% (165) | 75.0% (24) | 0.95 | (0.58, 1.54) | 0.828 |
| Timing of baricitinib initiation - within the first 5 days of admission | 84.8% (165) | 81.1% (37) | 0.67 | (0.43, 1.05) | 0.082 |
| Timing of baricitinib initiation - within the first 7 days of admission | 84.8% (165) | 80.0% (45) | 0.69 | (0.46, 1.02) | 0.065 |
| Administration route of dexamethasone - Oral | 100.0% (12) | 84.6% (13) | 0.39 | (0.13, 1.13) | 0.081 |
| Administration route of dexamethasone - Intravenous injection | 83.7% (153) | 81.0% (63) | 0.95 | (0.62, 1.45) | 0.809 |
| Dosage of dexamethasone - Up to 6mg daily | 83.9% (62) | 87.1% (31) | 0.55 | (0.33, 0.92) | 0.023 |
| Dosage of dexamethasone - More than 6mg daily | 85.4% (103) | 77.8% (45) | 1.12 | (0.65, 1.91) | 0.686 |
| Dosage of tocilizumab - Up to 400mg | 83.1% (71) | 81.6% (76) | 0.81 | (0.52, 1.26) | 0.348 |
| Dosage of tocilizumab - More than 400mg | 86.2% (94) | 81.6% (76) | 0.91 | (0.57, 1.43) | 0.668 |
| Dosage of baricitinib - Up to 4mg daily | 84.8% (165) | 55.6% (18) | 0.74 | (0.40, 1.38) | 0.344 |
| Dosage of baricitinib - More than 4mg daily | 84.8% (165) | 89.7% (58) | 0.87 | (0.56, 1.37) | 0.549 |
| Without mechanical ventilation or ECMO | 84.9% (146) | 80.9% (68) | 0.81 | (0.52, 1.27) | 0.355 |
| With mechanical ventilation or ECMO | 84.2% (19) | 87.5% (8) | 2.46 | (0.79, 7.72) | 0.117 |
| With supplemental oxygen without ventilation | 82.8% (99) | 76.7% (43) | 0.94 | (0.56, 1.58) | 0.818 |
| Without ICU admission | 85.1% (74) | 78.1% (32) | 0.62 | (0.33, 1.17) | 0.140 |
| With ICU admission | 84.6% (91) | 84.1% (44) | 0.89 | (0.55, 1.46) | 0.656 |
| Receipt of interferon-beta-1b | 85.8% (148) | 79.0% (62) | 0.98 | (0.62, 1.55) | 0.935 |
| Receipt of ribavirin | 89.5% (57) | 82.8% (29) | 1.23 | (0.63, 2.39) | 0.539 |
| Receipt of tocilizumab or baricitinib | 84.8% (165) | 81.6% (76) | 0.86 | (0.57, 1.29) | 0.459 |
| **Hospital discharge (score ≤ 3)** |  |  |  |  |  |
| Overall | 80.6% (165) | 78.9% (76) | 0.85 | (0.57, 1.27) | 0.418 |
| Age ≤ 65 | 93.0% (71) | 96.3% (27) | 0.70 | (0.43, 1.14) | 0.154 |
| Age > 65 | 71.3% (94) | 69.4% (49) | 0.84 | (0.51, 1.40) | 0.510 |
| Male | 81.1% (106) | 75.6% (45) | 1.02 | (0.60, 1.74) | 0.939 |
| Female | 79.7% (59) | 83.9% (31) | 0.56 | (0.34, 0.93) | 0.025 |
| Timing of dexamethasone initiation - within the first 3 days of admission | 82.4% (108) | 70.5% (44) | 0.79 | (0.49, 1.28) | 0.333 |
| Timing of dexamethasone initiation - within the first 5 days of admission | 82.4% (142) | 74.1% (54) | 0.78 | (0.51, 1.19) | 0.244 |
| Timing of dexamethasone initiation - within the first 7 days of admission | 82.2% (157) | 77.0% (61) | 0.88 | (0.56, 1.38) | 0.572 |
| Timing of tocilizumab initiation - within the first 3 days of admission | 80.0% (60) | 78.9% (76) | 0.69 | (0.43, 1.09) | 0.109 |
| Timing of tocilizumab initiation - within the first 5 days of admission | 80.2% (86) | 78.9% (76) | 0.84 | (0.55, 1.30) | 0.438 |
| Timing of tocilizumab initiation - within the first 7 days of admission | 82.5% (120) | 78.9% (76) | 0.85 | (0.57, 1.28) | 0.443 |
| Timing of baricitinib initiation - within the first 3 days of admission | 80.6% (165) | 70.8% (24) | 1.03 | (0.61, 1.76) | 0.907 |
| Timing of baricitinib initiation - within the first 5 days of admission | 80.6% (165) | 78.4% (37) | 0.69 | (0.43, 1.13) | 0.142 |
| Timing of baricitinib initiation - within the first 7 days of admission | 80.6% (165) | 77.8% (45) | 0.69 | (0.45, 1.06) | 0.088 |
| Administration route of dexamethasone - Oral | 100.0% (12) | 84.6% (13) | 0.39 | (0.13, 1.13) | 0.081 |
| Administration route of dexamethasone - Intravenous injection | 79.1% (153) | 77.8% (63) | 0.94 | (0.62, 1.42) | 0.763 |
| Dosage of dexamethasone - Up to 6mg daily | 79.0% (62) | 87.1% (31) | 0.45 | (0.26, 0.77) | 0.004 |
| Dosage of dexamethasone - More than 6mg daily | 81.6% (103) | 73.3% (45) | 1.21 | (0.72, 2.04) | 0.472 |
| Dosage of tocilizumab - Up to 400mg | 80.3% (71) | 78.9% (76) | 0.95 | (0.63, 1.44) | 0.819 |
| Dosage of tocilizumab - More than 400mg | 80.9% (94) | 78.9% (76) | 0.79 | (0.51, 1.23) | 0.300 |
| Dosage of baricitinib - Up to 4mg daily | 80.6% (165) | 55.6% (18) | 0.62 | (0.33, 1.16) | 0.131 |
| Dosage of baricitinib - More than 4mg daily | 80.6% (165) | 86.2% (58) | 0.88 | (0.57, 1.36) | 0.560 |
| Without mechanical ventilation or ECMO | 82.9% (146) | 79.4% (68) | 0.84 | (0.55, 1.29) | 0.435 |
| With mechanical ventilation or ECMO | 63.2% (19) | 75.0% (8) | 0.61 | (0.21, 1.82) | 0.363 |
| With supplemental oxygen without ventilation | 79.8% (99) | 74.4% (43) | 1.00 | (0.61, 1.63) | 0.996 |
| Without ICU admission | 85.1% (74) | 78.1% (32) | 0.62 | (0.33, 1.17) | 0.140 |
| With ICU admission | 76.9% (91) | 79.5% (44) | 0.89 | (0.55, 1.46) | 0.652 |
| Receipt of interferon-beta-1b | 82.4% (148) | 75.8% (62) | 0.98 | (0.63, 1.53) | 0.931 |
| Receipt of ribavirin | 84.2% (57) | 82.8% (29) | 1.16 | (0.59, 2.27) | 0.670 |
| Receipt of tocilizumab or baricitinib | 80.6% (165) | 78.9% (76) | 0.85 | (0.57, 1.27) | 0.418 |
| **Recovery (score ≤ 4)** |  |  |  |  |  |
| Overall | 75.4% (118) | 68.6% (51) | 1.04 | (0.64, 1.67) | 0.883 |
| Age ≤ 65 | 87.2% (47) | 94.1% (17) | 0.73 | (0.41, 1.32) | 0.296 |
| Age > 65 | 67.6% (71) | 55.9% (34) | 1.04 | (0.58, 1.87) | 0.890 |
| Male | 76.9% (78) | 66.7% (30) | 1.42 | (0.79, 2.58) | 0.243 |
| Female | 72.5% (40) | 71.4% (21) | 0.54 | (0.27, 1.06) | 0.071 |
| Timing of dexamethasone initiation - within the first 3 days of admission | 78.2% (78) | 60.0% (30) | 1.09 | (0.58, 2.02) | 0.792 |
| Timing of dexamethasone initiation - within the first 5 days of admission | 76.9% (104) | 62.2% (37) | 1.02 | (0.59, 1.77) | 0.936 |
| Timing of dexamethasone initiation - within the first 7 days of admission | 77.0% (113) | 67.4% (43) | 1.08 | (0.65, 1.80) | 0.765 |
| Timing of tocilizumab initiation - within the first 3 days of admission | 71.7% (46) | 68.6% (51) | 0.79 | (0.46, 1.35) | 0.388 |
| Timing of tocilizumab initiation - within the first 5 days of admission | 72.1% (61) | 68.6% (51) | 1.01 | (0.60, 1.71) | 0.955 |
| Timing of tocilizumab initiation - within the first 7 days of admission | 76.7% (86) | 68.6% (51) | 1.03 | (0.63, 1.67) | 0.920 |
| Timing of baricitinib initiation - within the first 3 days of admission | 75.4% (118) | 52.9% (17) | 1.38 | (0.64, 2.94) | 0.405 |
| Timing of baricitinib initiation - within the first 5 days of admission | 75.4% (118) | 66.7% (24) | 1.01 | (0.54, 1.89) | 0.982 |
| Timing of baricitinib initiation - within the first 7 days of admission | 75.4% (118) | 67.7% (31) | 0.90 | (0.53, 1.52) | 0.682 |
| Administration route of dexamethasone - Oral | 100.0% (10) | 50.0% (4) | 0.93 | (0.09, 9.43) | 0.947 |
| Administration route of dexamethasone - Intravenous injection | 73.1% (108) | 70.2% (47) | 1.01 | (0.62, 1.65) | 0.966 |
| Dosage of dexamethasone - Up to 6mg daily | 75.5% (49) | 77.8% (18) | 0.59 | (0.30, 1.16) | 0.126 |
| Dosage of dexamethasone - More than 6mg daily | 75.4% (69) | 63.6% (33) | 1.32 | (0.72, 2.41) | 0.370 |
| Dosage of tocilizumab - Up to 400mg | 78.4% (51) | 68.6% (51) | 1.21 | (0.74, 2.00) | 0.446 |
| Dosage of tocilizumab - More than 400mg | 73.1% (67) | 68.6% (51) | 0.93 | (0.54, 1.60) | 0.795 |
| Dosage of baricitinib - Up to 4mg daily | 75.4% (118) | 42.9% (14) | 0.58 | (0.25, 1.34) | 0.201 |
| Dosage of baricitinib - More than 4mg daily | 75.4% (118) | 78.4% (37) | 1.12 | (0.68, 1.86) | 0.658 |
| Without mechanical ventilation or ECMO | 77.8% (99) | 69.8% (43) | 1.05 | (0.63, 1.74) | 0.864 |
| With mechanical ventilation or ECMO | 63.2% (19) | 62.5% (8) | 0.70 | (0.21, 2.31) | 0.546 |
| With supplemental oxygen without ventilation | 77.8% (99) | 69.8% (43) | 1.05 | (0.63, 1.74) | 0.864 |
| Without ICU admission | 77.8% (27) | 42.9% (7) | 1.73 | (0.59, 5.13) | 0.308 |
| With ICU admission | 74.7% (91) | 72.7% (44) | 0.94 | (0.57, 1.57) | 0.823 |
| Receipt of interferon-beta-1b | 79.0% (105) | 67.4% (46) | 1.20 | (0.73, 1.97) | 0.476 |
| Receipt of ribavirin | 77.8% (45) | 76.2% (21) | 1.16 | (0.54, 2.53) | 0.697 |
| Receipt of tocilizumab or baricitinib | 75.4% (118) | 68.6% (51) | 1.04 | (0.64, 1.67) | 0.883 |
| **Viral clearance (first negative PCR result)** |  |  |  |  |  |
| Overall | 47.7% (155) | 33.3% (72) | 1.94 | (1.01, 3.73) | 0.048 |
| Age ≤ 65 | 45.6% (68) | 34.6% (26) | 1.94 | (0.79, 4.78) | 0.147 |
| Age > 65 | 49.4% (87) | 32.6% (46) | 1.78 | (0.76, 4.16) | 0.179 |
| Male | 47.1% (102) | 31.0% (42) | 2.43 | (1.01, 5.84) | 0.047 |
| Female | 49.1% (53) | 36.7% (30) | 1.20 | (0.54, 2.67) | 0.654 |
| Timing of dexamethasone initiation - within the first 3 days of admission | 49.1% (106) | 30.2% (43) | 1.78 | (0.83, 3.84) | 0.140 |
| Timing of dexamethasone initiation - within the first 5 days of admission | 47.1% (136) | 32.1% (53) | 1.59 | (0.80, 3.16) | 0.188 |
| Timing of dexamethasone initiation - within the first 7 days of admission | 46.4% (151) | 33.3% (60) | 1.85 | (0.90, 3.81) | 0.095 |
| Timing of tocilizumab initiation - within the first 3 days of admission | 52.5% (59) | 33.3% (72) | 2.38 | (1.22, 4.63) | 0.011 |
| Timing of tocilizumab initiation - within the first 5 days of admission | 47.1% (85) | 33.3% (72) | 2.25 | (1.11, 4.55) | 0.025 |
| Timing of tocilizumab initiation - within the first 7 days of admission | 47.1% (119) | 33.3% (72) | 2.10 | (1.06, 4.19) | 0.035 |
| Timing of baricitinib initiation - within the first 3 days of admission | 47.7% (155) | 33.3% (24) | 1.61 | (0.64, 4.03) | 0.309 |
| Timing of baricitinib initiation - within the first 5 days of admission | 47.7% (155) | 29.7% (37) | 1.71 | (0.77, 3.81) | 0.187 |
| Timing of baricitinib initiation - within the first 7 days of admission | 47.7% (155) | 28.9% (45) | 1.96 | (0.90, 4.29) | 0.090 |
| Administration route of dexamethasone - Oral | 66.7% (12) | 38.5% (13) | 0.79 | (0.15, 4.23) | 0.773 |
| Administration route of dexamethasone - Intravenous injection | 46.2% (143) | 32.2% (59) | 2.27 | (1.11, 4.63) | 0.025 |
| Dosage of dexamethasone - Up to 6mg daily | 53.6% (56) | 40.0% (30) | 1.34 | (0.59, 3.09) | 0.480 |
| Dosage of dexamethasone - More than 6mg daily | 44.4% (99) | 28.6% (42) | 2.35 | (0.93, 5.92) | 0.070 |
| Dosage of tocilizumab - Up to 400mg | 40.9% (66) | 33.3% (72) | 1.10 | (0.50, 2.41) | 0.816 |
| Dosage of tocilizumab - More than 400mg | 52.8% (89) | 33.3% (72) | 2.70 | (1.41, 5.19) | 0.003 |
| Dosage of baricitinib - Up to 4mg daily | 47.7% (155) | 33.3% (15) | 0.98 | (0.36, 2.71) | 0.971 |
| Dosage of baricitinib - More than 4mg daily | 47.7% (155) | 33.3% (57) | 2.21 | (1.07, 4.53) | 0.032 |
| Without mechanical ventilation or ECMO | 48.6% (140) | 32.8% (64) | 2.12 | (1.05, 4.31) | 0.037 |
| With mechanical ventilation or ECMO | 40.0% (15) | 37.5% (8) | 0.97 | (0.25, 3.74) | 0.965 |
| With supplemental oxygen without ventilation | 55.3% (94) | 42.5% (40) | 2.19 | (0.96, 4.99) | 0.062 |
| Without ICU admission | 39.4% (71) | 16.1% (31) | 2.37 | (0.81, 6.93) | 0.113 |
| With ICU admission | 54.8% (84) | 46.3% (41) | 1.76 | (0.78, 4.00) | 0.173 |
| Receipt of interferon-beta-1b | 48.6% (140) | 34.5% (58) | 2.25 | (1.09, 4.66) | 0.029 |
| Receipt of ribavirin | 42.6% (54) | 28.6% (28) | 1.67 | (0.53, 5.25) | 0.376 |
| Receipt of tocilizumab or baricitinib | 47.7% (155) | 33.3% (72) | 1.94 | (1.01, 3.73) | 0.048 |
| **Low viral load (Ct value ≥ 35)** |  |  |  |  |  |
| Overall | 46.8% (156) | 35.2% (71) | 1.49 | (0.85, 2.60) | 0.162 |
| Age ≤ 65 | 44.8% (67) | 30.8% (26) | 2.02 | (0.81, 5.08) | 0.132 |
| Age > 65 | 48.3% (89) | 37.8% (45) | 1.29 | (0.65, 2.53) | 0.466 |
| Male | 46.5% (101) | 47.6% (42) | 1.26 | (0.65, 2.45) | 0.494 |
| Female | 47.3% (55) | 17.2% (29) | 3.16 | (1.09, 9.10) | 0.034 |
| Timing of dexamethasone initiation - within the first 3 days of admission | 43.3% (104) | 40.0% (40) | 1.07 | (0.56, 2.07) | 0.827 |
| Timing of dexamethasone initiation - within the first 5 days of admission | 45.5% (134) | 40.0% (50) | 1.07 | (0.58, 1.97) | 0.823 |
| Timing of dexamethasone initiation - within the first 7 days of admission | 47.7% (149) | 38.6% (57) | 1.29 | (0.72, 2.29) | 0.392 |
| Timing of tocilizumab initiation - within the first 3 days of admission | 44.1% (59) | 35.2% (71) | 1.40 | (0.76, 2.57) | 0.279 |
| Timing of tocilizumab initiation - within the first 5 days of admission | 43.5% (85) | 35.2% (71) | 1.46 | (0.77, 2.76) | 0.248 |
| Timing of tocilizumab initiation - within the first 7 days of admission | 44.9% (118) | 35.2% (71) | 1.41 | (0.78, 2.52) | 0.250 |
| Timing of baricitinib initiation - within the first 3 days of admission | 46.8% (156) | 41.7% (24) | 1.35 | (0.64, 2.81) | 0.428 |
| Timing of baricitinib initiation - within the first 5 days of admission | 46.8% (156) | 37.1% (35) | 1.21 | (0.60, 2.45) | 0.597 |
| Timing of baricitinib initiation - within the first 7 days of admission | 46.8% (156) | 34.9% (43) | 1.47 | (0.70, 3.09) | 0.308 |
| Administration route of dexamethasone - Oral | 50.0% (12) | 8.3% (12) | NA | NA | NA |
| Administration route of dexamethasone - Intravenous injection | 46.5% (144) | 40.7% (59) | 1.46 | (0.84, 2.57) | 0.182 |
| Dosage of dexamethasone - Up to 6mg daily | 54.4% (57) | 37.9% (29) | 1.56 | (0.64, 3.79) | 0.321 |
| Dosage of dexamethasone - More than 6mg daily | 42.4% (99) | 33.3% (42) | 1.36 | (0.69, 2.69) | 0.368 |
| Dosage of tocilizumab - Up to 400mg | 43.3% (67) | 35.2% (71) | 1.03 | (0.53, 2.02) | 0.922 |
| Dosage of tocilizumab - More than 400mg | 49.4% (89) | 35.2% (71) | 1.92 | (1.10, 3.34) | 0.021 |
| Dosage of baricitinib - Up to 4mg daily | 46.8% (156) | 25.0% (16) | 2.20 | (0.57, 8.49) | 0.252 |
| Dosage of baricitinib - More than 4mg daily | 46.8% (156) | 38.2% (55) | 1.40 | (0.78, 2.51) | 0.255 |
| Without mechanical ventilation or ECMO | 47.1% (140) | 33.3% (63) | 1.57 | (0.85, 2.89) | 0.146 |
| With mechanical ventilation or ECMO | 43.8% (16) | 50.0% (8) | 0.95 | (0.32, 2.81) | 0.920 |
| With supplemental oxygen without ventilation | 46.9% (96) | 51.3% (39) | 1.07 | (0.54, 2.12) | 0.843 |
| Without ICU admission | 45.7% (70) | 6.5% (31) | 5.51 | (0.95, 31.77) | 0.056 |
| With ICU admission | 47.7% (86) | 57.5% (40) | 0.90 | (0.46, 1.75) | 0.749 |
| Receipt of interferon-beta-1b | 48.2% (139) | 38.6% (57) | 1.36 | (0.76, 2.45) | 0.299 |
| Receipt of ribavirin | 42.6% (54) | 40.0% (25) | 1.20 | (0.51, 2.81) | 0.676 |
| Receipt of tocilizumab or baricitinib | 46.8% (156) | 35.2% (71) | 1.49 | (0.85, 2.60) | 0.162 |
| **IgG antibody** |  |  |  |  |  |
| Overall | 85.5% (138) | 84.9% (53) | 0.97 | (0.61, 1.54) | 0.909 |
| Age ≤ 65 | 93.1% (58) | 100.0% (18) | 0.41 | (0.20, 0.85) | 0.016 |
| Age > 65 | 80.0% (80) | 77.1% (35) | 1.25 | (0.70, 2.21) | 0.445 |
| Male | 84.4% (90) | 83.3% (30) | 1.05 | (0.59, 1.89) | 0.860 |
| Female | 87.5% (48) | 87.0% (23) | 0.76 | (0.39, 1.46) | 0.398 |
| Timing of dexamethasone initiation - within the first 3 days of admission | 86.0% (93) | 76.5% (34) | 1.16 | (0.62, 2.17) | 0.631 |
| Timing of dexamethasone initiation - within the first 5 days of admission | 87.3% (118) | 80.0% (40) | 1.15 | (0.65, 2.03) | 0.620 |
| Timing of dexamethasone initiation - within the first 7 days of admission | 87.0% (131) | 82.2% (45) | 1.00 | (0.60, 1.64) | 0.985 |
| Timing of tocilizumab initiation - within the first 3 days of admission | 85.2% (54) | 84.9% (53) | 0.89 | (0.56, 1.43) | 0.637 |
| Timing of tocilizumab initiation - within the first 5 days of admission | 84.4% (77) | 84.9% (53) | 0.95 | (0.59, 1.54) | 0.831 |
| Timing of tocilizumab initiation - within the first 7 days of admission | 86.7% (105) | 84.9% (53) | 0.96 | (0.60, 1.55) | 0.877 |
| Timing of baricitinib initiation - within the first 3 days of admission | 85.5% (138) | 68.2% (22) | 1.92 | (0.94, 3.92) | 0.072 |
| Timing of baricitinib initiation - within the first 5 days of admission | 85.5% (138) | 78.8% (33) | 0.97 | (0.51, 1.86) | 0.926 |
| Timing of baricitinib initiation - within the first 7 days of admission | 85.5% (138) | 82.1% (39) | 0.95 | (0.55, 1.65) | 0.868 |
| Administration route of dexamethasone - Oral | 81.8% (11) | 100.0% (12) | 0.49 | (0.18, 1.37) | 0.162 |
| Administration route of dexamethasone - Intravenous injection | 85.8% (127) | 80.5% (41) | 1.13 | (0.66, 1.91) | 0.658 |
| Dosage of dexamethasone - Up to 6mg daily | 82.4% (51) | 95.8% (24) | 0.74 | (0.35, 1.59) | 0.437 |
| Dosage of dexamethasone - More than 6mg daily | 87.4% (87) | 75.9% (29) | 1.19 | (0.67, 2.11) | 0.544 |
| Dosage of tocilizumab - Up to 400mg | 80.6% (62) | 84.9% (53) | 1.13 | (0.72, 1.79) | 0.593 |
| Dosage of tocilizumab - More than 400mg | 89.5% (76) | 84.9% (53) | 0.88 | (0.54, 1.41) | 0.581 |
| Dosage of baricitinib - Up to 4mg daily | 85.5% (138) | 76.9% (13) | 1.40 | (0.78, 2.49) | 0.256 |
| Dosage of baricitinib - More than 4mg daily | 85.5% (138) | 87.5% (40) | 0.88 | (0.51, 1.50) | 0.625 |
| Without mechanical ventilation or ECMO | 86.6% (127) | 83.7% (49) | 1.01 | (0.62, 1.63) | 0.978 |
| With mechanical ventilation or ECMO | 72.7% (11) | 100.0% (4) | 0.43 | (0.11, 1.65) | 0.196 |
| With supplemental oxygen without ventilation | 85.9% (85) | 86.2% (29) | 0.92 | (0.50, 1.72) | 0.800 |
| Without ICU admission | 81.5% (65) | 76.9% (26) | 1.19 | (0.61, 2.30) | 0.607 |
| With ICU admission | 89.0% (73) | 92.6% (27) | 0.70 | (0.36, 1.39) | 0.311 |
| Receipt of interferon-beta-1b | 84.1% (126) | 85.4% (41) | 0.79 | (0.47, 1.32) | 0.363 |
| Receipt of ribavirin | 93.0% (43) | 93.8% (16) | 1.23 | (0.66, 2.29) | 0.502 |
| Receipt of tocilizumab or baricitinib | 85.5% (138) | 84.9% (53) | 0.97 | (0.61, 1.54) | 0.909 |
| **In-hospital death or mechanical ventilation (score ≥ 7) or intensive care unit admission** | | | | | |
| Overall | 33.6% (137) | 27.3% (66) | 0.96 | (0.47, 2.00) | 0.922 |
| Age ≤ 65 | 20.3% (59) | 4.0% (25) | 0.00 | (0.00, 0.00) | <0.001 |
| Age > 65 | 43.6% (78) | 41.5% (41) | 0.89 | (0.41, 1.94) | 0.771 |
| Male | 32.6% (89) | 30.8% (39) | 0.97 | (0.38, 2.46) | 0.942 |
| Female | 35.4% (48) | 22.2% (27) | 0.90 | (0.31, 2.63) | 0.847 |
| Timing of dexamethasone initiation - within the first 3 days of admission | 31.0% (87) | 39.5% (38) | 0.76 | (0.33, 1.74) | 0.518 |
| Timing of dexamethasone initiation - within the first 5 days of admission | 31.9% (116) | 35.6% (45) | 0.80 | (0.38, 1.68) | 0.547 |
| Timing of dexamethasone initiation - within the first 7 days of admission | 32.1% (131) | 32.7% (52) | 0.75 | (0.35, 1.61) | 0.461 |
| Timing of tocilizumab initiation - within the first 3 days of admission | 32.5% (40) | 27.3% (66) | 1.46 | (0.55, 3.83) | 0.442 |
| Timing of tocilizumab initiation - within the first 5 days of admission | 29.2% (65) | 27.3% (66) | 0.95 | (0.37, 2.46) | 0.916 |
| Timing of tocilizumab initiation - within the first 7 days of admission | 33.0% (97) | 27.3% (66) | 1.03 | (0.46, 2.32) | 0.949 |
| Timing of baricitinib initiation - within the first 3 days of admission | 33.6% (137) | 42.9% (21) | 0.75 | (0.31, 1.80) | 0.515 |
| Timing of baricitinib initiation - within the first 5 days of admission | 33.6% (137) | 34.4% (32) | 0.98 | (0.43, 2.23) | 0.959 |
| Timing of baricitinib initiation - within the first 7 days of admission | 33.6% (137) | 33.3% (39) | 1.08 | (0.48, 2.41) | 0.858 |
| Administration route of dexamethasone - Oral | 8.3% (12) | 15.4% (13) | NA | NA | NA |
| Administration route of dexamethasone - Intravenous injection | 36.0% (125) | 30.2% (53) | 1.06 | (0.47, 2.35) | 0.892 |
| Dosage of dexamethasone - Up to 6mg daily | 39.6% (48) | 13.8% (29) | 2.26 | (0.67, 7.65) | 0.188 |
| Dosage of dexamethasone - More than 6mg daily | 30.3% (89) | 37.8% (37) | 0.75 | (0.31, 1.84) | 0.526 |
| Dosage of tocilizumab - Up to 400mg | 34.3% (67) | 27.3% (66) | 0.74 | (0.34, 1.60) | 0.443 |
| Dosage of tocilizumab - More than 400mg | 32.9% (70) | 27.3% (66) | 1.29 | (0.55, 3.05) | 0.556 |
| Dosage of baricitinib - Up to 4mg daily | 33.6% (137) | 43.8% (16) | 0.55 | (0.22, 1.33) | 0.183 |
| Dosage of baricitinib - More than 4mg daily | 33.6% (137) | 22.0% (50) | 1.20 | (0.50, 2.89) | 0.679 |
| Without mechanical ventilation or ECMO | 33.6% (137) | 27.3% (66) | 0.96 | (0.47, 2.00) | 0.922 |
| With mechanical ventilation or ECMO | NA (0) | NA (0) | NA | NA | NA |
| With supplemental oxygen without ventilation | 44.4% (90) | 36.6% (41) | 0.78 | (0.36, 1.69) | 0.532 |
| Without ICU admission | 17.6% (74) | 21.9% (32) | 1.10 | (0.33, 3.71) | 0.876 |
| With ICU admission | 52.4% (63) | 32.4% (34) | 0.97 | (0.38, 2.42) | 0.940 |
| Receipt of interferon-beta-1b | 31.1% (122) | 34.0% (53) | 0.75 | (0.35, 1.61) | 0.460 |
| Receipt of ribavirin | 24.5% (49) | 20.0% (25) | 0.55 | (0.16, 1.92) | 0.343 |
| Receipt of tocilizumab or baricitinib | 33.6% (137) | 27.3% (66) | 0.96 | (0.47, 2.00) | 0.922 |
| **In-hospital death (score = 10)** |  |  |  |  |  |
| Overall | 17.6% (165) | 19.7% (76) | 0.63 | (0.29, 1.35) | 0.233 |
| Age ≤ 65 | 5.6% (71) | 3.7% (27) | NA | NA | NA |
| Age > 65 | 26.6% (94) | 28.6% (49) | 0.73 | (0.32, 1.69) | 0.460 |
| Male | 17.0% (106) | 22.2% (45) | 0.67 | (0.25, 1.79) | 0.416 |
| Female | 18.6% (59) | 16.1% (31) | 0.60 | (0.20, 1.85) | 0.373 |
| Timing of dexamethasone initiation - within the first 3 days of admission | 15.7% (108) | 27.3% (44) | 0.31 | (0.14, 0.72) | 0.006 |
| Timing of dexamethasone initiation - within the first 5 days of admission | 15.5% (142) | 24.1% (54) | 0.35 | (0.16, 0.74) | 0.007 |
| Timing of dexamethasone initiation - within the first 7 days of admission | 15.9% (157) | 21.3% (61) | 0.51 | (0.22, 1.15) | 0.105 |
| Timing of tocilizumab initiation - within the first 3 days of admission | 16.7% (60) | 19.7% (76) | 0.51 | (0.19, 1.38) | 0.185 |
| Timing of tocilizumab initiation - within the first 5 days of admission | 17.4% (86) | 19.7% (76) | 0.54 | (0.22, 1.30) | 0.168 |
| Timing of tocilizumab initiation - within the first 7 days of admission | 15.0% (120) | 19.7% (76) | 0.48 | (0.21, 1.09) | 0.079 |
| Timing of baricitinib initiation - within the first 3 days of admission | 17.6% (165) | 25.0% (24) | 0.51 | (0.18, 1.41) | 0.193 |
| Timing of baricitinib initiation - within the first 5 days of admission | 17.6% (165) | 18.9% (37) | 0.67 | (0.26, 1.75) | 0.409 |
| Timing of baricitinib initiation - within the first 7 days of admission | 17.6% (165) | 20.0% (45) | 0.62 | (0.25, 1.53) | 0.302 |
| Administration route of dexamethasone - Oral | 0.0% (12) | 15.4% (13) | NA | NA | NA |
| Administration route of dexamethasone - Intravenous injection | 19.0% (153) | 20.6% (63) | 0.76 | (0.34, 1.69) | 0.494 |
| Dosage of dexamethasone - Up to 6mg daily | 19.4% (62) | 12.9% (31) | 0.70 | (0.18, 2.74) | 0.606 |
| Dosage of dexamethasone - More than 6mg daily | 16.5% (103) | 24.4% (45) | 0.55 | (0.22, 1.41) | 0.210 |
| Dosage of tocilizumab - Up to 400mg | 18.3% (71) | 19.7% (76) | 0.64 | (0.26, 1.57) | 0.327 |
| Dosage of tocilizumab - More than 400mg | 17.0% (94) | 19.7% (76) | 0.60 | (0.26, 1.41) | 0.238 |
| Dosage of baricitinib - Up to 4mg daily | 17.6% (165) | 44.4% (18) | 0.18 | (0.08, 0.42) | <0.001 |
| Dosage of baricitinib - More than 4mg daily | 17.6% (165) | 12.1% (58) | 1.14 | (0.45, 2.91) | 0.778 |
| Without mechanical ventilation or ECMO | 15.1% (146) | 19.1% (68) | 0.59 | (0.25, 1.39) | 0.228 |
| With mechanical ventilation or ECMO | 36.8% (19) | 25.0% (8) | 1.02 | (0.25, 4.07) | 0.980 |
| With supplemental oxygen without ventilation | 17.2% (99) | 23.3% (43) | 0.60 | (0.23, 1.57) | 0.292 |
| Without ICU admission | 14.9% (74) | 21.9% (32) | 0.40 | (0.13, 1.29) | 0.123 |
| With ICU admission | 19.8% (91) | 18.2% (44) | 0.84 | (0.31, 2.31) | 0.734 |
| Receipt of interferon-beta-1b | 16.9% (148) | 22.6% (62) | 0.51 | (0.23, 1.13) | 0.096 |
| Receipt of ribavirin | 15.8% (57) | 17.2% (29) | 1.00 | (0.27, 3.72) | 0.996 |
| Receipt of tocilizumab or baricitinib | 17.6% (165) | 19.7% (76) | 0.63 | (0.29, 1.35) | 0.233 |
| **Severe liver injury** |  |  |  |  |  |
| Overall | 18.5% (162) | 13.2% (76) | 1.15 | (0.43, 3.08) | 0.778 |
| Age ≤ 65 | 11.4% (70) | 14.8% (27) | 0.69 | (0.18, 2.70) | 0.588 |
| Age > 65 | 23.9% (92) | 12.2% (49) | 1.47 | (0.41, 5.35) | 0.554 |
| Male | 19.2% (104) | 13.3% (45) | 0.85 | (0.27, 2.72) | 0.788 |
| Female | 17.2% (58) | 12.9% (31) | 2.34 | (0.65, 8.43) | 0.189 |
| Timing of dexamethasone initiation - within the first 3 days of admission | 19.8% (106) | 13.6% (44) | 1.83 | (0.63, 5.33) | 0.267 |
| Timing of dexamethasone initiation - within the first 5 days of admission | 18.0% (139) | 16.7% (54) | 1.38 | (0.56, 3.39) | 0.477 |
| Timing of dexamethasone initiation - within the first 7 days of admission | 18.2% (154) | 16.4% (61) | 0.91 | (0.34, 2.43) | 0.850 |
| Timing of tocilizumab initiation - within the first 3 days of admission | 23.7% (59) | 13.2% (76) | 1.47 | (0.45, 4.84) | 0.523 |
| Timing of tocilizumab initiation - within the first 5 days of admission | 20.0% (85) | 13.2% (76) | 1.22 | (0.41, 3.58) | 0.722 |
| Timing of tocilizumab initiation - within the first 7 days of admission | 18.6% (118) | 13.2% (76) | 1.10 | (0.39, 3.11) | 0.850 |
| Timing of baricitinib initiation - within the first 3 days of admission | 18.5% (162) | 20.8% (24) | 1.21 | (0.39, 3.70) | 0.740 |
| Timing of baricitinib initiation - within the first 5 days of admission | 18.5% (162) | 16.2% (37) | 1.42 | (0.51, 3.95) | 0.502 |
| Timing of baricitinib initiation - within the first 7 days of admission | 18.5% (162) | 15.6% (45) | 1.55 | (0.59, 4.05) | 0.370 |
| Administration route of dexamethasone - Oral | 25.0% (12) | 0.0% (13) | 0.00 | (0.00, 0.00) | <0.001 |
| Administration route of dexamethasone - Intravenous injection | 18.0% (150) | 15.9% (63) | 1.02 | (0.39, 2.66) | 0.960 |
| Dosage of dexamethasone - Up to 6mg daily | 20.0% (60) | 16.1% (31) | 1.23 | (0.35, 4.32) | 0.748 |
| Dosage of dexamethasone - More than 6mg daily | 17.6% (102) | 11.1% (45) | 1.07 | (0.30, 3.84) | 0.911 |
| Dosage of tocilizumab - Up to 400mg | 17.4% (69) | 13.2% (76) | 1.02 | (0.31, 3.29) | 0.979 |
| Dosage of tocilizumab - More than 400mg | 19.4% (93) | 13.2% (76) | 1.21 | (0.43, 3.39) | 0.710 |
| Dosage of baricitinib - Up to 4mg daily | 18.5% (162) | 16.7% (18) | 1.20 | (0.33, 4.31) | 0.780 |
| Dosage of baricitinib - More than 4mg daily | 18.5% (162) | 12.1% (58) | 1.17 | (0.39, 3.46) | 0.779 |
| Without mechanical ventilation or ECMO | 16.0% (144) | 13.2% (68) | 0.92 | (0.32, 2.69) | 0.884 |
| With mechanical ventilation or ECMO | 38.9% (18) | 12.5% (8) | NA | NA | NA |
| With supplemental oxygen without ventilation | 18.4% (98) | 18.6% (43) | 0.72 | (0.25, 2.10) | 0.550 |
| Without ICU admission | 9.7% (72) | 6.3% (32) | 2.61 | (0.44, 15.51) | 0.289 |
| With ICU admission | 25.6% (90) | 18.2% (44) | NA | (0.35, 2.79) | 0.979 |
| Receipt of interferon-beta-1b | 17.9% (145) | 9.7% (62) | 1.35 | (0.40, 4.49) | 0.625 |
| Receipt of ribavirin | 14.5% (55) | 6.9% (29) | 0.92 | (0.18, 4.74) | 0.917 |
| Receipt of tocilizumab or baricitinib | 18.5% (162) | 13.2% (76) | 1.15 | (0.43, 3.08) | 0.778 |
| **Acute renal failure** |  |  |  |  |  |
| Overall | 11.6% (164) | 5.3% (76) | 2.33 | (0.61, 8.82) | 0.213 |
| Age ≤ 65 | 8.5% (71) | 0.0% (27) | NA | NA | NA |
| Age > 65 | 14.0% (93) | 8.2% (49) | 1.27 | (0.35, 4.66) | 0.712 |
| Male | 12.4% (105) | 4.4% (45) | 3.74 | (0.63, 22.16) | 0.145 |
| Female | 10.2% (59) | 6.5% (31) | 0.80 | (0.16, 4.05) | 0.788 |
| Timing of dexamethasone initiation - within the first 3 days of admission | 13.0% (108) | 9.1% (44) | 1.52 | (0.39, 5.89) | 0.543 |
| Timing of dexamethasone initiation - within the first 5 days of admission | 12.1% (141) | 7.4% (54) | 1.53 | (0.41, 5.64) | 0.522 |
| Timing of dexamethasone initiation - within the first 7 days of admission | 10.9% (156) | 6.6% (61) | 1.83 | (0.47, 7.11) | 0.379 |
| Timing of tocilizumab initiation - within the first 3 days of admission | 20.0% (60) | 5.3% (76) | 2.18 | (0.59, 8.03) | 0.241 |
| Timing of tocilizumab initiation - within the first 5 days of admission | 18.6% (86) | 5.3% (76) | 3.55 | (0.91, 13.88) | 0.069 |
| Timing of tocilizumab initiation - within the first 7 days of admission | 13.4% (119) | 5.3% (76) | 2.69 | (0.68, 10.66) | 0.159 |
| Timing of baricitinib initiation - within the first 3 days of admission | 11.6% (164) | 12.5% (24) | 1.32 | (0.31, 5.58) | 0.703 |
| Timing of baricitinib initiation - within the first 5 days of admission | 11.6% (164) | 8.1% (37) | 1.71 | (0.43, 6.91) | 0.447 |
| Timing of baricitinib initiation - within the first 7 days of admission | 11.6% (164) | 6.7% (45) | 2.16 | (0.52, 8.99) | 0.287 |
| Administration route of dexamethasone - Oral | 0.0% (12) | 0.0% (13) | NA | NA | NA |
| Administration route of dexamethasone - Intravenous injection | 12.5% (152) | 6.3% (63) | 2.43 | (0.64, 9.21) | 0.190 |
| Dosage of dexamethasone - Up to 6mg daily | 13.1% (61) | 3.2% (31) | NA | NA | NA |
| Dosage of dexamethasone - More than 6mg daily | 10.7% (103) | 6.7% (45) | 1.18 | (0.29, 4.85) | 0.817 |
| Dosage of tocilizumab - Up to 400mg | 12.7% (71) | 5.3% (76) | 1.63 | (0.44, 6.07) | 0.461 |
| Dosage of tocilizumab - More than 400mg | 10.8% (93) | 5.3% (76) | 2.86 | (0.64, 12.78) | 0.167 |
| Dosage of baricitinib - Up to 4mg daily | 11.6% (164) | 11.1% (18) | 0.61 | (0.12, 3.04) | 0.548 |
| Dosage of baricitinib - More than 4mg daily | 11.6% (164) | 3.4% (58) | 3.81 | (0.68, 21.43) | 0.128 |
| Without mechanical ventilation or ECMO | 9.0% (145) | 4.4% (68) | 3.16 | (0.68, 14.75) | 0.143 |
| With mechanical ventilation or ECMO | 31.6% (19) | 12.5% (8) | NA | NA | NA |
| With supplemental oxygen without ventilation | 13.3% (98) | 7.0% (43) | 3.10 | (0.67, 14.38) | 0.147 |
| Without ICU admission | 0.0% (74) | 0.0% (32) | NA | NA | NA |
| With ICU admission | 21.1% (90) | 9.1% (44) | 2.39 | (0.64, 8.92) | 0.193 |
| Receipt of interferon-beta-1b | 11.6% (147) | 4.8% (62) | 2.59 | (0.61, 11.05) | 0.198 |
| Receipt of ribavirin | 10.5% (57) | 0.0% (29) | 0.00 | (0.00, 0.00) | <0.001 |
| Receipt of tocilizumab or baricitinib | 11.6% (164) | 5.3% (76) | 2.33 | (0.61, 8.82) | 0.213 |
| **Hyperinflammatory syndrome** |  |  |  |  |  |
| Overall | 88.9% (18) | 64.3% (14) | 2.32 | (0.87, 6.25) | 0.091 |
| Age ≤ 65 | 88.9% (9) | 50.0% (6) | 4.19 | (0.69, 25.50) | 0.110 |
| Age > 65 | 88.9% (9) | 75.0% (8) | 1.70 | (0.56, 5.18) | 0.321 |
| Male | 100.0% (9) | 71.4% (7) | 2.04 | (0.41, 10.26) | 0.355 |
| Female | 77.8% (9) | 57.1% (7) | 2.54 | (0.67, 9.64) | 0.155 |
| Timing of dexamethasone initiation - within the first 3 days of admission | 93.3% (15) | 66.7% (9) | 2.21 | (0.72, 6.74) | 0.154 |
| Timing of dexamethasone initiation - within the first 5 days of admission | 88.9% (18) | 66.7% (12) | 2.15 | (0.78, 5.90) | 0.131 |
| Timing of dexamethasone initiation - within the first 7 days of admission | 88.9% (18) | 69.2% (13) | 2.20 | (0.82, 5.90) | 0.112 |
| Timing of tocilizumab initiation - within the first 3 days of admission | 100.0% (15) | 64.3% (14) | 3.13 | (0.97, 10.13) | 0.057 |
| Timing of tocilizumab initiation - within the first 5 days of admission | 100.0% (16) | 64.3% (14) | 3.22 | (0.98, 10.63) | 0.054 |
| Timing of tocilizumab initiation - within the first 7 days of admission | 88.9% (18) | 64.3% (14) | 2.32 | (0.87, 6.25) | 0.091 |
| Timing of baricitinib initiation - within the first 3 days of admission | 88.9% (18) | 75.0% (8) | 1.83 | (0.61, 5.47) | 0.266 |
| Timing of baricitinib initiation - within the first 5 days of admission | 88.9% (18) | 60.0% (10) | 2.13 | (0.71, 6.33) | 0.166 |
| Timing of baricitinib initiation - within the first 7 days of admission | 88.9% (18) | 63.6% (11) | 2.02 | (0.74, 5.56) | 0.163 |
| Administration route of dexamethasone - Oral | NA% (0) | 50.0% (6) | NA | NA | NA |
| Administration route of dexamethasone - Intravenous injection | 88.9% (18) | 75.0% (8) | 1.76 | (0.61, 5.11) | 0.282 |
| Dosage of dexamethasone - Up to 6mg daily | 87.5% (8) | 71.4% (7) | 1.60 | (0.48, 5.33) | 0.411 |
| Dosage of dexamethasone - More than 6mg daily | 90.0% (10) | 57.1% (7) | 3.20 | (0.65, 15.83) | 0.141 |
| Dosage of tocilizumab - Up to 400mg | 71.4% (7) | 64.3% (14) | 1.44 | (0.42, 4.89) | 0.537 |
| Dosage of tocilizumab - More than 400mg | 100.0% (11) | 64.3% (14) | 3.03 | (0.90, 10.24) | 0.072 |
| Dosage of baricitinib - Up to 4mg daily | 88.9% (18) | 75.0% (4) | 1.73 | (0.36, 8.20) | 0.469 |
| Dosage of baricitinib - More than 4mg daily | 88.9% (18) | 60.0% (10) | 2.60 | (0.85, 8.01) | 0.092 |
| Without mechanical ventilation or ECMO | 87.5% (16) | 64.3% (14) | 2.25 | (0.84, 6.02) | 0.101 |
| With mechanical ventilation or ECMO | 100.0% (2) | NA% (0) | 0.00 | (0.00, 0.00) | <0.001 |
| With supplemental oxygen without ventilation | 100.0% (9) | 60.0% (5) | 2.85 | (0.55, 14.68) | 0.185 |
| Without ICU admission | 77.8% (9) | 63.6% (11) | 1.78 | (0.55, 5.78) | 0.314 |
| With ICU admission | 100.0% (9) | 66.7% (3) | 6.08 | (0.86, 43.17) | 0.067 |
| Receipt of interferon-beta-1b | 100.0% (14) | 80.0% (5) | 2.09 | (0.45, 9.63) | 0.318 |
| Receipt of ribavirin | 100.0% (1) | 100.0% (2) | 0.00 | (0.00, 0.00) | <0.001 |
| Receipt of tocilizumab or baricitinib | 88.9% (18) | 64.3% (14) | 2.32 | (0.87, 6.25) | 0.091 |
| **Secondary infection** |  |  |  |  |  |
| Overall | 7.4% (163) | 2.6% (76) | 2.97 | (0.62, 14.31) | 0.173 |
| Age ≤ 65 | 7.2% (69) | 3.7% (27) | NA | NA | NA |
| Age > 65 | 7.4% (94) | 2.0% (49) | NA | NA | NA |
| Male | 7.7% (104) | 2.2% (45) | NA | NA | NA |
| Female | 6.8% (59) | 3.2% (31) | NA | NA | NA |
| Timing of dexamethasone initiation - within the first 3 days of admission | 7.4% (108) | 4.5% (44) | 1.61 | (0.33, 7.95) | 0.558 |
| Timing of dexamethasone initiation - within the first 5 days of admission | 7.8% (141) | 3.7% (54) | 2.02 | (0.42, 9.81) | 0.380 |
| Timing of dexamethasone initiation - within the first 7 days of admission | 7.7% (155) | 3.3% (61) | 2.66 | (0.54, 13.04) | 0.227 |
| Timing of tocilizumab initiation - within the first 3 days of admission | 8.3% (60) | 2.6% (76) | 3.49 | (0.59, 20.60) | 0.166 |
| Timing of tocilizumab initiation - within the first 5 days of admission | 8.1% (86) | 2.6% (76) | 3.48 | (0.65, 18.73) | 0.145 |
| Timing of tocilizumab initiation - within the first 7 days of admission | 7.5% (120) | 2.6% (76) | 2.98 | (0.60, 14.79) | 0.180 |
| Timing of baricitinib initiation - within the first 3 days of admission | 7.4% (163) | 4.2% (24) | NA | NA | NA |
| Timing of baricitinib initiation - within the first 5 days of admission | 7.4% (163) | 5.4% (37) | 1.32 | (0.26, 6.62) | 0.732 |
| Timing of baricitinib initiation - within the first 7 days of admission | 7.4% (163) | 4.4% (45) | 1.71 | (0.35, 8.24) | 0.503 |
| Administration route of dexamethasone - Oral | 8.3% (12) | 0.0% (13) | NA | NA | NA |
| Administration route of dexamethasone - Intravenous injection | 7.3% (151) | 3.2% (63) | 2.85 | (0.57, 14.27) | 0.202 |
| Dosage of dexamethasone - Up to 6mg daily | 4.9% (61) | 3.2% (31) | NA | NA | NA |
| Dosage of dexamethasone - More than 6mg daily | 8.8% (102) | 2.2% (45) | NA | NA | NA |
| Dosage of tocilizumab - Up to 400mg | 1.4% (71) | 2.6% (76) | NA | NA | NA |
| Dosage of tocilizumab - More than 400mg | 12.0% (92) | 2.6% (76) | 4.71 | (0.95, 23.31) | 0.058 |
| Dosage of baricitinib - Up to 4mg daily | 7.4% (163) | 0.0% (18) | NA | NA | NA |
| Dosage of baricitinib - More than 4mg daily | 7.4% (163) | 3.4% (58) | 2.61 | (0.53, 12.94) | 0.239 |
| Without mechanical ventilation or ECMO | 7.6% (144) | 2.9% (68) | 2.78 | (0.57, 13.66) | 0.207 |
| With mechanical ventilation or ECMO | 5.3% (19) | 0.0% (8) | NA | NA | NA |
| With supplemental oxygen without ventilation | 11.3% (97) | 4.7% (43) | 2.84 | (0.57, 14.10) | 0.200 |
| Without ICU admission | 0.0% (74) | 0.0% (32) | NA | NA | NA |
| With ICU admission | 13.5% (89) | 4.5% (44) | 3.13 | (0.64, 15.38) | 0.159 |
| Receipt of interferon-beta-1b | 5.5% (146) | 3.2% (62) | 2.09 | (0.41, 10.79) | 0.376 |
| Receipt of ribavirin | 9.1% (55) | 0.0% (29) | NA | NA | NA |
| Receipt of tocilizumab or baricitinib | 7.4% (163) | 2.6% (76) | 2.97 | (0.62, 14.31) | 0.173 |
| **Thrombotic and bleeding events** |  |  |  |  |  |
| Overall | 10.6% (141) | 4.9% (41) | 1.39 | (0.32, 6.00) | 0.658 |
| Age ≤ 66 | 8.5% (59) | 0.0% (16) | NA | NA | NA |
| Age > 66 | 12.2% (82) | 8.0% (25) | 0.93 | (0.21, 4.15) | 0.928 |
| Male | 11.2% (89) | 8.7% (23) | 0.87 | (0.19, 3.89) | 0.849 |
| Female | 9.6% (52) | 0.0% (18) | NA | NA | NA |
| Timing of dexamethasone initiation - within the first 3 days of admission | 11.6% (95) | 7.4% (27) | 1.11 | (0.25, 4.99) | 0.890 |
| Timing of dexamethasone initiation - within the first 5 days of admission | 12.4% (121) | 6.3% (32) | 1.35 | (0.31, 5.84) | 0.684 |
| Timing of dexamethasone initiation - within the first 7 days of admission | 11.3% (133) | 5.9% (34) | 1.23 | (0.28, 5.34) | 0.779 |
| Timing of tocilizumab initiation - within the first 3 days of admission | 14.8% (54) | 4.9% (41) | 2.19 | (0.47, 10.28) | 0.316 |
| Timing of tocilizumab initiation - within the first 5 days of admission | 12.0% (75) | 4.9% (41) | 1.71 | (0.37, 7.82) | 0.489 |
| Timing of tocilizumab initiation - within the first 7 days of admission | 13.3% (105) | 4.9% (41) | 1.80 | (0.41, 7.79) | 0.430 |
| Timing of baricitinib initiation - within the first 3 days of admission | 10.6% (141) | 11.8% (17) | 0.56 | (0.14, 2.20) | 0.400 |
| Timing of baricitinib initiation - within the first 5 days of admission | 10.6% (141) | 8.0% (25) | 0.91 | (0.22, 3.87) | 0.900 |
| Timing of baricitinib initiation - within the first 7 days of admission | 10.6% (141) | 6.9% (29) | 1.05 | (0.24, 4.52) | 0.952 |
| Administration route of dexamethasone - Oral | 0.0% (10) | 0.0% (8) | NA | NA | NA |
| Administration route of dexamethasone - Intravenous injection | 11.5% (131) | 6.1% (33) | 1.24 | (0.29, 5.25) | 0.766 |
| Dosage of dexamethasone - Up to 6mg daily | 14.8% (54) | 5.6% (18) | NA | NA | NA |
| Dosage of dexamethasone - More than 6mg daily | 8.0% (87) | 4.3% (23) | NA | NA | NA |
| Dosage of tocilizumab - Up to 400mg | 9.7% (62) | 4.9% (41) | 1.16 | (0.24, 5.70) | 0.852 |
| Dosage of tocilizumab - More than 400mg | 11.4% (79) | 4.9% (41) | 1.57 | (0.34, 7.27) | 0.563 |
| Dosage of baricitinib - Up to 4mg daily | 10.6% (141) | 0.0% (11) | NA | NA | NA |
| Dosage of baricitinib - More than 4mg daily | 10.6% (141) | 6.7% (30) | 1.01 | (0.24, 4.36) | 0.985 |
| Without mechanical ventilation or ECMO | 10.2% (128) | 5.0% (40) | 1.27 | (0.29, 5.61) | 0.747 |
| With mechanical ventilation or ECMO | 15.4% (13) | 0.0% (1) | NA | NA | NA |
| With supplemental oxygen without ventilation | 15.9% (82) | 5.0% (20) | NA | NA | NA |
| Without ICU admission | 0.0% (70) | 4.2% (24) | NA | NA | NA |
| With ICU admission | 21.1% (71) | 5.9% (17) | NA | NA | NA |
| Receipt of interferon-beta-1b | 10.2% (127) | 3.4% (29) | NA | NA | NA |
| Receipt of ribavirin | 8.7% (46) | 0.0% (11) | NA | NA | NA |
| Receipt of tocilizumab or baricitinib | 10.6% (141) | 4.9% (41) | 1.39 | (0.32, 6.00) | 0.658 |
|  |  |  |  |  |  |

Note:

CI = confidence interval; Ct = cycle threshold; ECMO = extracorporeal membrane oxygenation; HR = hazard ratio; ICU = intensive care unit; IgG = immunoglobulin G; PCR = polymerase chain reaction

† HR >1 (or <1) indicates that tocilizumab use was associated with better (worse) clinical improvement, earlier (later) hospital discharge or recovery, or higher (lower) risk of adverse clinical outcomes compared to that of baricitinib

Supplementary Table 6. Comparison of laboratory parameters of patients in tocilizumab and baricitinib groups from baseline to last measurement during hospitalization

|  |  | Baseline | Baseline (with last measurement) | Last measurement | Paired difference | P-value* for difference = 0 |
| --- | --- | --- | --- | --- | --- | --- |
| White blood cell, ×10^9^/L (Mean±SD)  [normal range: 3.7-9.2 ×10^9^/L] | Tocilizumab | 7.7 (3.7) | 7.7 (3.7) | 8.5 (6.0) | 0.8 (6.6) | 0.262 |
|  | Baricitinib | 7.3 (4.0) | 7.3 (4.0) | 9.7 (5.2) | 2.3 (4.9) | <0.001 |
|  | P-value† | 0.646 | 0.646 | 0.177 | 0.133 |  |
|  |  |  |  |  |  |  |
| Neutrophil, ×10^9^/L (Mean±SD)  [normal range: 1.7-5.8 ×10^9^/L] | Tocilizumab | 6.3 (3.6) | 6.3 (3.6) | 6.2 (5.8) | -0.1 (6.4) | 0.857 |
|  | Baricitinib | 6.2 (3.8) | 6.2 (3.8) | 7.7 (5.1) | 1.5 (5.0) | 0.045 |
|  | P-value† | 0.845 | 0.848 | 0.089 | 0.115 |  |
|  |  |  |  |  |  |  |
| Lymphocyte, ×10^9^/L (Mean±SD)  [normal range: 1.0-3.1 ×10^9^/L] | Tocilizumab | 0.8 (0.5) | 0.8 (0.5) | 1.5 (0.7) | 0.7 (0.7) | <0.001 |
|  | Baricitinib | 0.7 (0.5) | 0.7 (0.5) | 1.3 (0.8) | 0.5 (0.7) | <0.001 |
|  | P-value† | 0.315 | 0.316 | 0.059 | 0.195 |  |
|  |  |  |  |  |  |  |
| Platelet, ×10^9^/L (Mean±SD)  [normal range: 145-370 ×10^9^/L] | Tocilizumab | 186.0 (76.9) | 186.0 (76.9) | 280.0 (112.2) | 94.0 (118.0) | <0.001 |
|  | Baricitinib | 187.7 (65.7) | 187.7 (65.7) | 288.1 (102.2) | 100.4 (94.4) | <0.001 |
|  | P-value† | 0.901 | 0.901 | 0.638 | 0.728 |  |
|  |  |  |  |  |  |  |
| Lactate dehydrogenase, U/L (Mean±SD)  [normal range: 110-210 U/L] | Tocilizumab | 402.1 (166.8) | 402.1 (166.8) | 440.2 (1,549.4) | 38.1 (1,544.7) | 0.709 |
|  | Baricitinib | 408.2 (139.1) | 412.0 (137.9) | 347.5 (273.4) | -64.5 (264.3) | 0.112 |
|  | P-value† | 0.808 | 0.697 | 0.406 | 0.350 |  |
|  |  |  |  |  |  |  |
| Creatine kinase, U/L (Mean±SD)  [normal range: 26-192 U/L] | Tocilizumab | 265.4 (501.9) | 274.1 (508.3) | 126.8 (390.0) | -147.3 (561.8) | 0.006 |
|  | Baricitinib | 225.1 (296.4) | 195.3 (257.2) | 143.5 (703.5) | -51.8 (742.8) | 0.484 |
|  | P-value† | 0.508 | 0.162 | 0.821 | 0.291 |  |
|  |  |  |  |  |  |  |
| Total bilirubin, μmol/L (Mean±SD)  [normal range: 5-27 μmol/L] | Tocilizumab | 10.3 (5.8) | 10.3 (5.8) | 11.3 (19.1) | 1.0 (19.1) | 0.476 |
|  | Baricitinib | 10.8 (8.4) | 10.8 (8.4) | 14.3 (23.7) | 3.5 (17.2) | 0.232 |
|  | P-value† | 0.730 | 0.730 | 0.485 | 0.446 |  |
|  |  |  |  |  |  |  |
| C-reactive protein, mg/L (Mean±SD)  [normal range: <5 mg/L] | Tocilizumab | 75.2 (61.0) | 75.2 (61.0) | 17.2 (39.0) | -58.0 (70.0) | <0.001 |
|  | Baricitinib | 81.7 (63.8) | 80.4 (63.3) | 15.8 (27.0) | -64.6 (70.8) | <0.001 |
|  | P-value† | 0.604 | 0.683 | 0.776 | 0.634 |  |
|  |  |  |  |  |  |  |
| Ferritin, pmol/L (Mean±SD)  [normal range: <1573 pmol/L] | Tocilizumab | 4,126.6 (4,362.0) | 4,316.3 (4,391.3) | 2,487.8 (2,796.7) | -1,828.4 (3,649.6) | <0.001 |
|  | Baricitinib | 4,203.4 (4,098.7) | 3,881.8 (3,653.8) | 2,655.8 (2,403.1) | -1,225.9 (2,634.5) | 0.004 |
|  | P-value† | 0.924 | 0.579 | 0.731 | 0.287 |  |
|  |  |  |  |  |  |  |
| Cycle threshold value, cycle (Mean±SD) | Tocilizumab | 23.1 (5.2) | 23.1 (5.3) | 32.7 (5.8) | 9.6 (7.9) | <0.001 |
|  | Baricitinib | 23.0 (6.4) | 22.7 (6.3) | 30.3 (10.0) | 7.7 (10.0) | <0.001 |
|  | P-value† | 0.934 | 0.720 | 0.120 | 0.248 |  |
|  |  |  |  |  |  |  |
| eGFR, mL/min/1.73m^2^ (Mean±SD)  [normal range: >90 mL/min/1.73m^2^] | Tocilizumab | 98.6 (71.2) | 98.6 (71.2) | 114.2 (57.6) | 15.6 (81.1) | 0.014 |
|  | Baricitinib | 106.5 (42.9) | 106.5 (42.9) | 125.6 (370.7) | 19.1 (366.3) | 0.500 |
|  | P-value† | 0.349 | 0.349 | 0.697 | 0.903 |  |
|  |  |  |  |  |  |  |
| ALP, U/L (Mean±SD)  [normal range: 30-120 U/L] | Tocilizumab | 73.1 (32.2) | 73.1 (32.2) | 80.2 (39.6) | 7.1 (43.3) | 0.160 |
|  | Baricitinib | 67.5 (22.5) | 67.5 (22.5) | 82.5 (35.6) | 15.0 (31.7) | <0.001 |
|  | P-value† | 0.232 | 0.232 | 0.675 | 0.209 |  |
|  |  |  |  |  |  |  |
| ALT, U/L (Mean±SD)  [normal range: 7-50 U/L] | Tocilizumab | 44.9 (63.5) | 44.9 (63.5) | 109.6 (542.5) | 64.7 (543.2) | 0.068 |
|  | Baricitinib | 41.6 (30.7) | 41.6 (30.7) | 69.0 (225.4) | 27.5 (227.5) | 0.185 |
|  | P-value† | 0.566 | 0.566 | 0.321 | 0.362 |  |
|  |  |  |  |  |  |  |
| Hemoglobin, g/dL (Mean±SD)  [normal range: 13.4-17.1 g/dL] | Tocilizumab | 13.3 (2.0) | 13.3 (2.0) | 11.5 (2.2) | -1.8 (2.3) | <0.001 |
|  | Baricitinib | 13.0 (1.6) | 13.0 (1.6) | 12.2 (2.0) | -0.9 (1.8) | 0.003 |
|  | P-value† | 0.570 | 0.570 | 0.063 | 0.028 |  |

Notes:

ALP = alkaline phosphatase; ALT = alanine aminotransferase; eGFR = estimated glomerular filtration rate; SD = standard deviation

† P-value is calculated by linear regression between tocilizumab and baricitinib groups

* P-value is calculated by paired t-test within tocilizumab and baricitinib groups
